# Clonal selection in the human Vδ1 T cell repertoire indicates γδ TCR-dependent adaptive immune surveillance

**DOI:** 10.1038/ncomms14760

**Published:** 2017-03-01

**Authors:** Martin S. Davey, Carrie R. Willcox, Stephen P. Joyce, Kristin Ladell, Sofya A. Kasatskaya, James E. McLaren, Stuart Hunter, Mahboob Salim, Fiyaz Mohammed, David A. Price, Dmitriy M. Chudakov, Benjamin E. Willcox

**Affiliations:** 1 Cancer Immunology and Immunotherapy Centre, Institute for Immunology and Immunotherapy, University of Birmingham, Edgbaston, Birmingham B15 2TT, UK; 2 Division of Infection and Immunity, Cardiff University School of Medicine, Heath Park, Cardiff CF14 4XN, UK; 3 Shemyakin-Ovchinnikov Institute of Bioorganic Chemistry of the Russian Academy of Sciences, Moscow 117997, Russia; 4 Pirogov Russian National Research Medical University, Moscow 117997, Russia; 5 Central European Institute of Technology, Masaryk University, Brno 625 00, Czech Republic

## Abstract

γδ T cells are considered to be innate-like lymphocytes that respond rapidly to stress without clonal selection and differentiation. Here we use next-generation sequencing to probe how this paradigm relates to human Vδ2^neg^ T cells, implicated in responses to viral infection and cancer. The prevalent Vδ1 T cell receptor (TCR) repertoire is private and initially unfocused in cord blood, typically becoming strongly focused on a few high-frequency clonotypes by adulthood. Clonal expansions have differentiated from a naive to effector phenotype associated with CD27 downregulation, retaining proliferative capacity and TCR sensitivity, displaying increased cytotoxic markers and altered homing capabilities, and remaining relatively stable over time. Contrastingly, Vδ2^+^ T cells express semi-invariant TCRs, which are present at birth and shared between individuals. Human Vδ1^+^ T cells have therefore evolved a distinct biology from the Vδ2^+^ subset, involving a central, personalized role for the γδ TCR in directing a highly adaptive yet unconventional form of immune surveillance.

## Abstract (web summary)

γδ T cells are generally considered innate-like lymphocytes. Here the authors sequence human γδ T cell receptors (TCR) to show focusing of the private Vδ1 TCR repertoire, suggesting that, unlike Vδ2 T cells, the Vδ1 T cell compartment has adaptive attributes.

γδTcells have been preserved alongside αβ T cells and B cells over the last ∼450 million years of vertebrate evolution^1^, and are increasingly recognized as having important roles in immune responses to both microbial and non-microbial stress challenges^2^. Although γδ T cells recognize target cells in an MHC-independent fashion, consistent with a lack of surface CD4/CD8αβ co-receptor expression, the key paradigms underpinning their distinct immunobiology are unclear. Mouse studies have highlighted γδ T cell subsets bearing semi-invariant TCRs^1^,^3^,^4^,^5^, suggestive of an innate-like biology and a limited range of self-ligands. In humans the Vδ2^+^ repertoire predominant in peripheral blood arguably conforms to this paradigm. As for other unconventional lymphocyte populations in humans such as natural killer T (NKT) cells and mucosal-associated invariant T (MAIT) cells, Vδ2^+^ T cells feature a conserved chain pairing (in the case of Vδ2^+^ cells with Vγ9), restricted CDR3 region diversity, comprising motifs conserved between many individuals^6^, and is generated early in gestation^7^. Most importantly, there is strong evidence Vδ2^+^ cells share a conserved biology, as they display potent TCR-dependent reactivity to pyrophosphate antigens generated by many species of bacteria/mycobacteria, with the butyrophilin-like molecule BTN3A1 a central player in antigen recognition^8^.

The extent to which these ideas apply to the human Vδ2^neg^ repertoire, present in both blood and peripheral tissues, is unclear. Previous studies have highlighted a diverse variable (V) region usage for this subset, and a diverse range of ligands have also been proposed for γδ TCRs^3^, although to what extent they represent physiological reactivities is uncertain. Moreover, some studies have highlighted the potential for TCR-independent effector functions^9^,^10^. Nevertheless, Vδ2^neg^ γδ T cells are implicated in immune responses to viral infection, particularly cytomegalovirus (CMV)^11^,^12^, but also Epstein Barr virus^13^,^14^, and can also recognize a broad range of cancer cells^15^. One possibility is that within a seemingly diverse Vδ2^neg^ subset there exist conserved innate-like populations; however, a distinct biology underpinning Vδ2^neg^ γδ T cell stress responses might alternatively be involved. Characterizing the Vδ2^neg^ TCR repertoire would enhance our understanding of this area.

Here, we use next-generation sequencing (NGS) approaches to define the Vδ2^neg^ repertoire from human peripheral blood, focusing on the predominant Vδ1 subset. By comparing neonates and healthy CMV-seropositive/CMV-seronegative adults, we show that the Vδ1 TCR repertoire is private, initially unfocused, and subsequently shaped by TCR-dependent clonal selection, concurrent with differentiation. These findings suggest that a distinct mode of adaptive immune surveillance applies to the Vδ1 subset, and raise further questions regarding the nature of the γδ TCR-linked stress challenges driving evolution of these responses *in vivo*.

## Results

### Vδ2^+^ and Vδ2^neg^ T cell phenotype in human peripheral blood

**Figure 1:**
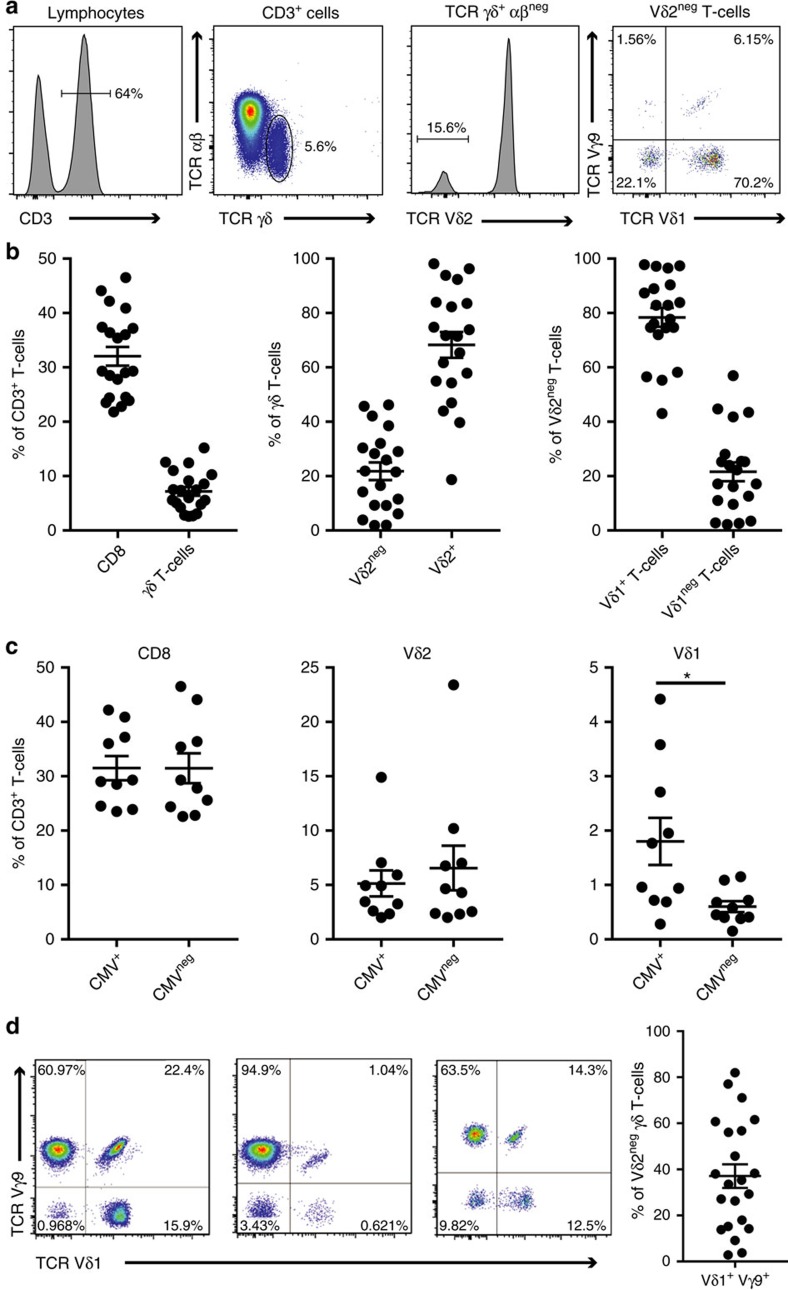
Peripheral blood γδ T-cell populations in donors analysed for γδ TCR repertoires. (**a**) Major peripheral blood γδ T-cell populations identified by γδ TCR chain specific antibodies. Flow cytometry plots are representative of 20 donors. (**b**) Frequency of γδ T cells in CD3^+^ lymphocytes (left graph), abundance of Vδ2^+^ and Vδ2^neg^ T cells in the γδ T cell compartment (Middle), and proportion of Vδ1^+^ T cells in the Vδ2^neg^ γδ T-cell sub-population (Right). Graphs show the mean±s.e.m. from 20 donors. (**c**) Effect of donor CMV-seropositivity on CD8^+^, Vδ2^+^ and Vδ1^+^ cell percentages in total T cells. Graphs show the mean±s.e.m. from 10 CMV-seropositive and 10 CMV-seronegative donors. (**d**) Prevalence of a Vγ9 chain pairing in Vδ1^+^ T cells. Graphs show the mean±s.e.m. and flow cytometry plots are representative of 20 donors. Data analysed by student's *t*-test, *P=0.0228.

We first characterized Vδ2^neg^ T cells in the peripheral blood of healthy adult donors selected for subsequent repertoire analyses. To account for possible effects of CMV status on phenotype^12^,^16^ we compared 10 CMV-seropositive and 10 CMV-seronegative donors, selecting individuals of a similar age (18–30 years), as previous studies have highlighted age-related changes in the CMV-specific αβ T-cell repertoire^17^,^18^ and the γδ T-cell repertoire in general^19^,^20^. In all individuals we detected Vδ2^+^ and Vδ2^neg^ γδ T-cell populations (Fig. 1a), the latter dominated by Vδ1^+^ cells (Fig. 1a,b). Consistent with previous studies, we observed a modestly increased proportion of Vδ1^+^ cells in CMV^+^ individuals (Fig. 1c). Furthermore, we noted the presence of a Vγ9/Vδ1 chain pairing in all individuals to varying degrees (Fig. 1d). Both Vδ2^neg^ T cells and the Vδ1^+^ subsets of γδ T cells displayed a mixed CD27^-^/CD45RA^+^ or CD27^+^/CD45RA^+^ phenotype in most individuals (Supplementary Fig. 1A,B), as previously described^16^. In comparison, Vδ2^+^ T cells were predominantly CD27^+^CD45RA^−^ (Supplementary Fig. 1A,B), consistent with previous studies^21^.

### The Vδ1 TCR repertoire is focused on dominant clonotypes

**Figure 2:**
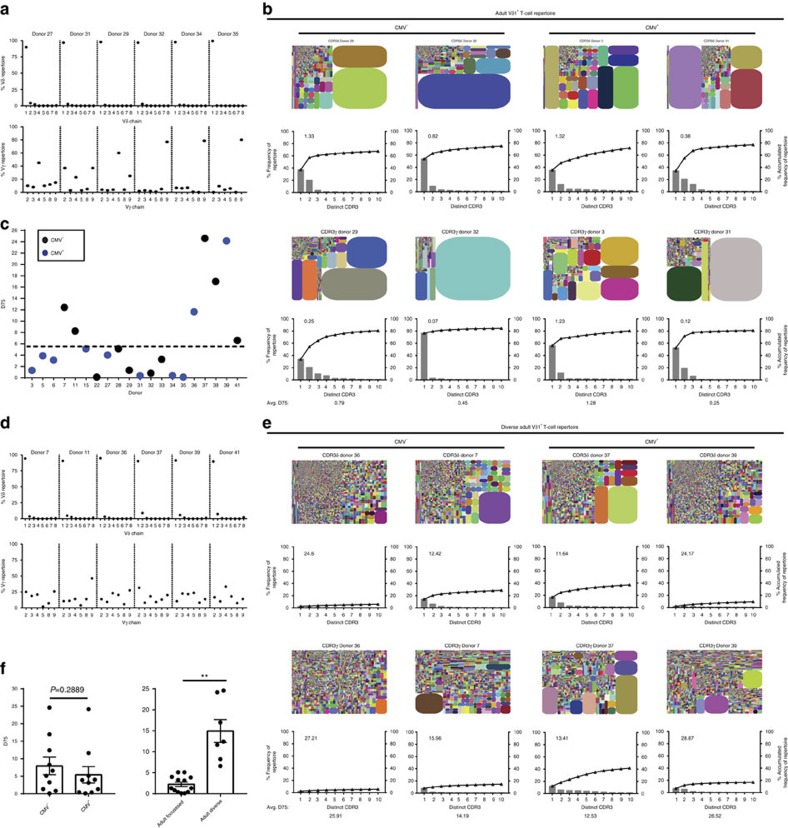
The Vδ1^+^ TCR repertoire is focused on a few dominant clonotypes in healthy adults. (**a**) Vγ and Vδ chain usage by γδ TCR sequences from sorted Vδ1^+^ T cells from peripheral blood. Graphs show six representative donors of 13. (**b**) Tree maps show CDR3 clonotype usage in relation to repertoire size (each CDR3 colour is chosen randomly and does not match between plots) and graphs show the individual clone frequency (left *y* axis) and the accumulated frequency for the first 10 most prevalent clonotypes (right *y* axis). (**c**) Analysis of inter-donor diversity by D75 (percentage of clonotypes required to occupy 75% of the total TCR repertoire) from TCRδ repertoire analyses from 20 donors with CMV-seropositive (blue dots), CMV-seronegative individuals (black dots) and lowest quartile range plotted (dashed line). (**d**) Vγ and Vδ chain usage and (**e**) Tree maps and accumulated frequency graphs, for γδ TCR repertoires in donors with a D75>6. (**f**) Comparison of mean ±s.e.m. of TCRδ D75 values for 10 CMV-seropositive and 10 CMV-seronegative donors (Left) and focused donors (*n*=13) against diverse donors (*n*=7) (Right). Data were analysed by student's *t*-test, **P=0.0002.

We used amplicon rescued multiplex (ARM)-PCR and NGS methods to analyse the Vδ2^neg^ TCR repertoire in healthy adults, focusing initially on the dominant Vδ1 subset. We achieved a mean sequencing depth of 346,371±27,993 reads with an average of 2,742±432 unique CDR3 sequences for TCRδ and 384,182±31,547 reads with an average of 1,261±170 unique CDR3 sequences for TCRγ (Supplementary Table 1), with no correlation observed between sequencing depth and numbers of unique CDR3 assigned (Supplementary Fig. 2A). Consistent with successful purification of Vδ1 T cells, nearly all TCRδ sequences obtained used Vδ1 (Fig. 2a); for TCRγ, consistent with flow cytometry (Fig. 1a,d), Vγ9 sequences were commonly prevalent (Fig. 2a) and correlated with Vγ9 antibody staining (Supplementary Fig. 2B), although clonotypes using other functional Vγ gene segments were detected in all donors (Fig. 2a; Supplementary Fig. 2C,D).

Strikingly, in most individuals (8/10 CMV^+^ and 5/10 CMV^neg^ adults), remarkably strong focusing of the Vδ1^+^ response was observed towards a small number of individual clonotypes (Fig. 2b; Supplementary Fig. 2E). This was evident for both TCRγ and TCRδ chains, and the majority of total error-corrected reads could be accounted for by a small number of the most prevalent clonotypes (≤10) (Fig. 2b). TCRδ D75 values (the percentage of unique sequences required to account for 75% of total reads) were <6% in this grouping (mean 2.23±s.e.m. 0.53%), emphasising extreme skewing (Fig. 2c). In addition, the Vγ9/Vδ1 populations consistently present were often highly clonally focused. To address whether this extreme clonotypic focusing was restricted to Vδ1 sequences, we developed a negative selection strategy enabling analysis of the broader Vδ2^neg^ population (Supplementary Fig. 3A,B), in four individuals. A more diverse range of Vδ sequences were observed in these analyses, including chiefly Vδ3 and Vδ8, with evidence of moderate clonotypic focusing in some individuals for Vδ2^neg^, Vδ1^neg^ Vδ chains (Supplementary Fig. 3C), although these Vδ regions were used in a small percentage of clonotypes in these donors.

In contrast with this ‘focused adult' subgroup, dramatically less focused repertoires were observed in seven individuals (TCRδ D75 mean 14.95±s.e.m. 2.74%; in most cases the top 10 sequences represented <20% of the total error-corrected reads detected) and used a varied set of Vγ gene segments (Fig. 2d,e; Supplementary Fig. 2D,F). These results establish that while not universal, extreme clonotypic focusing is a common feature of the Vδ1 and Vδ2^neg^ γδ TCR repertoire. Applying a TCRδ D75 cut-off threshold of 6% (equivalent to the lowest quartile TCRδ D75 values), we defined this minority of individuals with higher D75 values as a ‘diverse adult' subgroup (Fig. 2c). While the average TCRδ D75 values and Shannon-Weiner index for CDR3δ1 diversity highlighted a trend towards decreased diversity (lower TCRδ D75) in CMV^+^ versus CMV^neg^ donors, the difference was not significant (Fig. 2f, left; Supplementary Fig. 4). Notably the diverse adult donors were mostly (5/7) CMV seronegative (Fig. 2c), and included the youngest members of the cohort. The difference in mean TCRδ D75 values between adult focused and adult diverse donors was ∼7-fold (2.23±0.53 versus 14.95±2.74 respectively, *P*<0.0002; Student's *t*-test) (Fig. 2f, right).

### The cord blood Vδ1 TCR repertoire is unfocused

**Figure 3:**
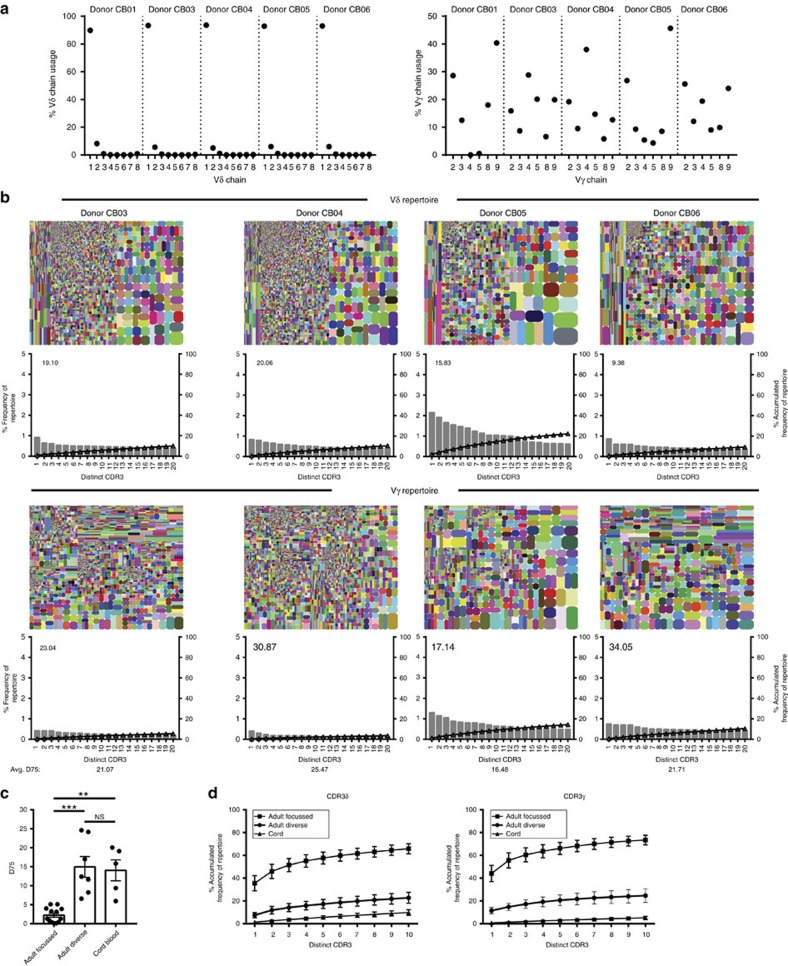
The cord blood Vδ1^+^ TCR repertoire is composed of an unfocused set of γδ TCRs. (**a**) Vγ and Vδ chain usage by sorted Vδ1^+^ T cells from cord blood samples. (**b**) Tree maps for both CDR3γ and CDR3δ clonotype usage in relation to repertoire size and accumulated frequency graphs for each of the top 20 most prevalent clonotypes. (**c**) Comparison of mean±s.e.m. of TCRδ D75 values from adult focused (*n*=13), adult unfocused (*n*=7) and cord blood (*n*=5) donors. (**d**) Accumulated frequencies means±s.e.m. occupied by the first 10 clonotypes for each donor and grouped into adult focused donors (*n*=13), adult diverse (*n*=7) and cord blood (*n*=5). Data were analysed by Kruskal–Wallis ANOVA with Dunn's post-test comparisons, **P=0.0049 and ***P=0.0007.

To investigate how the Vδ1 repertoire differed in early life, we carried out comparable TCR repertoire analyses on the Vδ1^+^ subpopulation of cord blood-purified γδ T cells (Fig. 3a,b). Vδ1^+^ cells dominate the cord blood γδ repertoire^7^, and expressed Vδ1 paired with diverse Vγ regions (Fig. 3a). Cord blood TCRδ1 and TCRγ CDR3 sequences were extremely unfocused (TCRδ D75 mean 14.06±s.e.m. 2.76%), in contrast to focused adult Vδ1 repertoires (mean 2.23±s.e.m. 0.53%), but similar to unfocused adult samples (mean 14.95±s.e.m. 2.74%) (Fig. 3c), and comprised numerous low frequency clonotypes, the most prevalent of which represented <1.30% and <2.17% of the total unique CDR3s detected, for TCRγ and TCRδ respectively (see cumulative frequency plots accounting for the 10 highest prevalence clonotypes, Fig. 3d).

### The Vδ1 TCR repertoire is dominated by private clonotypes

**Figure 4:**
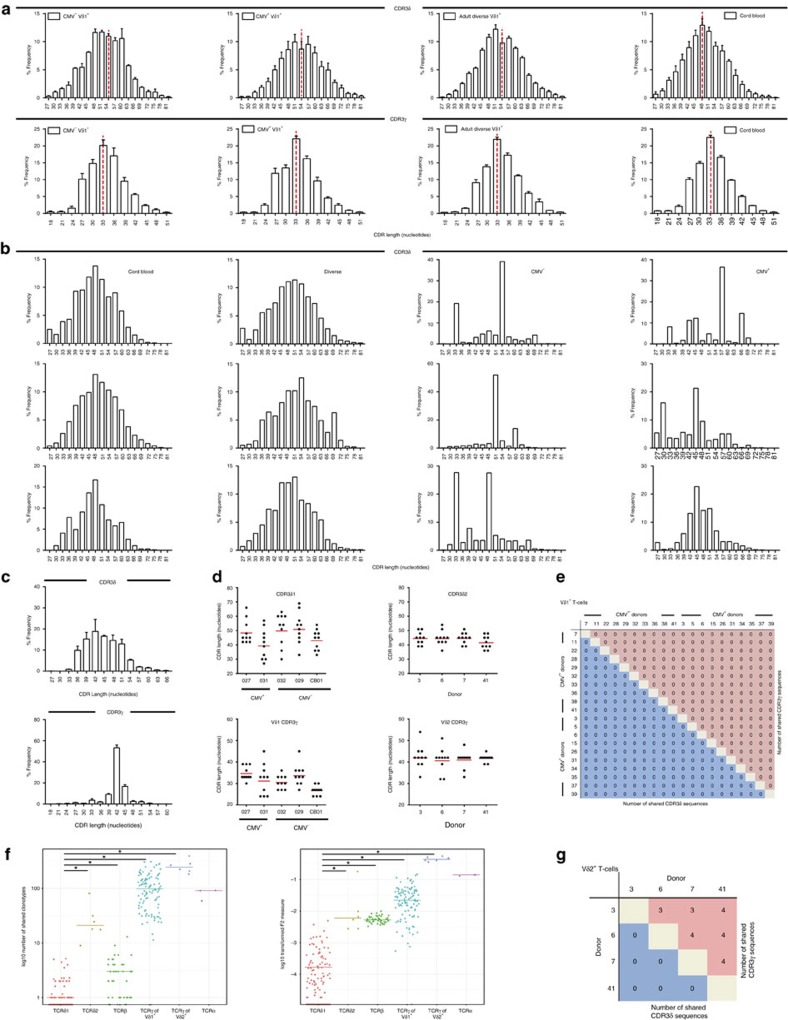
CDR3 length and diversity within the Vδ1 and Vδ2 TCR. (**a**) Comparison of the mean ±s.e.m. from frequency-normalized CDR3δ and CDR3γ length spectratyping for Vδ1^+^ T cells in CMV^neg^ (*n*=5), CMV^+^ (*n*=8), adult diverse (*n*=7) and cord blood (*n*=5) donors. (**b**) Comparison of non-normalized CDR3δ length spectratyping for Vδ1^+^ T cells from 3 representative individuals from CMV-seronegative (representative of *n*=5), CMV-seropositive (representative of *n*=8), adult diverse (representative of *n*=7) and cord blood (representative of *n*=5) donor groupings. (**c**) Non-normalized length spectratyping for CDR3δ and CDR3γ in adult Vδ2^+^ T cells (*n*=4). (**d**) Length distribution and mean (red line) within the top 10 most prevalent clonotypes in CDR3δ (left two panels) and CDR3γ (right two panels) of Vδ1^+^ T cells (5 donors shown, representative of 20) and Vδ2^+^ T cells (from 4 donors). (**e**) Publicity in the 10 most prevalent clonotypes for each donor's Vδ1^+^ TCR repertoire compared against all other donors (both aa and nt sequences compared). (**f**) Comparison of relative publicity in TCR repertoires. Overlap of individual TCR repertoires in TCRδ1 (*n*=15), TCRδ2 (*n*=4), TCRβ (*n*=15, ref PMID: 27183615), TCRγ of Vδ1^+^ cells, TCRγ of Vδ2^+^ cells and TCRα (*n*=3). Each dot shows relative similarity of repertoires for a pair of unrelated donors in terms of the shared TCR variants with identical amino acid CDR3 sequence, and V and J segments used. Overlap was estimated either as a number of clonotypes shared between the sets of top-1,000 largest clonotypes of each repertoire (left), or as a sum of clonotype frequencies shared between the total repertoires (F2 metrics of VDJTools software, right). (**g**) Publicity in the 10 most prevalent clonotypes for each donor's Vδ2^+^ TCR repertoire compared against all other donors (both aa and nt sequences compared). Mean overlap of sequences between each group was analysed by Kruskal–Wallis ANOVA with Dunn's post-test comparisons *P<0.003.

Detailed comparisons of CDR3 length (presented as spectratype plots) within and between individuals indicated that in Vδ1^+^ T cells, the mean CDR3δ1 length was substantially greater than CDR3γ (mean 54 versus 33 nucleotides (nt; Fig. 4a), as expected^22^). In addition, we detected a minority of exceedingly long Vδ1 CDR3s of >87 nt, some having up to 52 nontemplated (N) nt added, which appear to be *bona fide* productive TCR chains (Supplementary Fig. 5A,B). Frequency-normalized analyses of CDR3 lengths in focused adults, diverse adults and cord blood were essentially identical (Fig. 4a). However, non-normalized comparisons of CDR3 length distributions, taking into account the frequency of individual clonotypes, indicated that only unfocused adults' repertoires had similar profiles to cord blood. In contrast, individuals with focused Vδ1 TCR repertoires displayed highly skewed profiles (Fig. 4b).

Comparable analyses of the human Vδ2^+^ repertoire in four adults highlighted that while Vδ1^+^ cells used various Vγ and Jγ gene segments, Vδ2^+^ cells almost exclusively used Vγ9 paired with JγP (Supplementary Fig. 6A,B), with CDR3γ9 lengths of 11–18 amino acids (33–54 nt) (Fig. 4c), and >50% of CDR3s composed of 14 amino acids in all donors. Moreover, analysis of the ten most frequent TCRδ and TCRγ clonotypes in Vδ2^+^ cells from each donor revealed constrained lengths in both CDR3γ9 and CDR3δ2 (Fig. 4d). In contrast, in Vδ1^+^ cells the CDR3γ and CDR3δ lengths of the ten most frequent clonotypes, typically accounting for >50% of the repertoire, were extremely diverse both within and between individuals (Fig. 4d, Supplementary Fig. 6C). Consistent with this, multidimensional clustering analysis confirmed the TCRγ CDR3 length distributions of Vδ2^+^ T cells were similar across different donors, whereas those of the Vδ1 compartment were highly individual (Supplementary Fig. 6D).

Importantly, comparisons of the ten most prevalent TCRγ and TCRδ1 clonotypes from each donor revealed they were private sequences, absent in any other individuals either at a nucleotide or amino acid level (Supplementary Tables 2–5; Fig. 4e). Consistent with this, they frequently involved extremely complex CDR3 regions, comprising numerous N/P nucleotide additions (1–11 nt for TCRγ; 5–28 nt for TCRδ) in addition to D and J region nucleotides (Supplementary Tables 2–3), indicating rare recombination events. This was particularly pronounced for the predominant Vδ1 chains in each donor, which utilized 1–2 Dδ regions and contained a mean of 16 N/P nucleotides. Indeed, after error correction, nearly all CDR3δ and CDR3γ sequences were found to be private. The very few TCRγ and TCRδ clonotypes observed in >1 individual generally resulted from relatively simple recombination events with little N nt addition, and were low in frequency. Comparison of Vδ1 repertoire data with age and sex matched TCRβ repertoire data revealed that Vδ1 as a repertoire was even more private than TCRβ (Fig. 4f). Therefore, the Vδ1 TCR repertoire was overwhelmingly private, with different TCR clonotypes present in each individual, and these observations also extended to non-Vδ1 sequences in our Vδ2^neg^ γδ T cell analyses, suggesting it may be a generic property of the Vδ2^neg^ TCR repertoire.

In contrast, several Vδ2 subset-derived CDR3γ protein sequences were shared between all four samples at a relatively high frequency (Fig. 4g; Supplementary Fig. 7A,B). These involved recombination of Vγ9 and JγP segments with limited N/P nucleotide addition (generally <3 nt) indicating their origin from convergent recombination^23^. Vγ9 sequences in Vδ2^+^ cells were constrained in length (Fig. 4c,d) and were more public than TCRδ, TCRβ chains and TCRγ in Vδ1^+^ cells (Fig. 4f), and often contained a typical 14 amino acid sequence motif (Supplementary Fig. 7C)^6^,^7^,^24^. Although Vδ2 sequences were relatively private compared with TCRγ (Fig. 4f,g), more CDR3δ2 sequences were shared between donors than CDR3δ1 sequences (Fig. 4f). As previously noted, CDR3δ2 often contained a hydrophobic L or V residue at position 6 (Supplementary Fig. 7D)^24^. Therefore, in stark contrast to Vδ1^+^ T cells, but analogous to iNKT^25^ and MAIT^26^ populations, the Vδ2Vγ9 population expresses a semi-invariant TCR.

### Vδ1 clonal expansions are long-lived and differentiated

**Figure 5:**
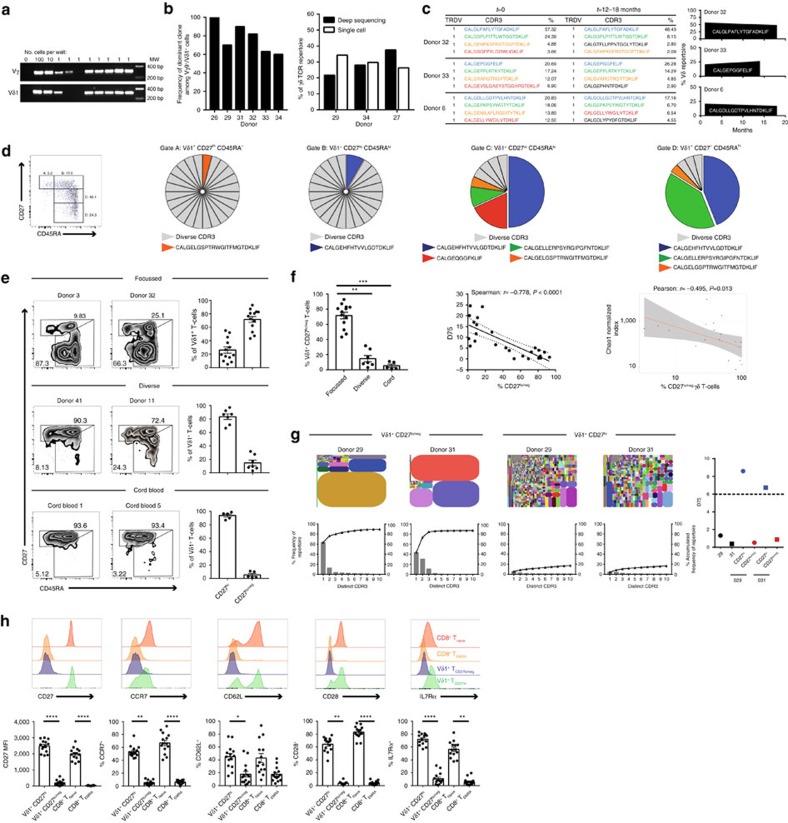
Vδ1 clonal expansion and differentiation is associated with decreased CD27 expression. (**a**) PCR detection of Vγ9 and Vδ1 TCR chains in single cell sorted Vγ9Vδ1 T cells. *n*=6. (**b**) Frequency of each individual's dominant clone among single cell sorted Vγ9Vδ1 T cells. (**c**) Sequential TCR repertoire analyses in three individuals using RACE-PCR (left panel, first time point), and deep-sequencing ARM-PCR (middle panel, second time point, 12–18 months later). Clonotypic sequences are coloured consistently. The right panel depicts the frequency of the top clone within the repertoire over the two time points. (**d**) Relationship between CD27 and CD45RA expression and clonality by single cell PCR analysis of CDR3δ. Each colour represents an individual CDR3δ, with clonal sequences labelled below each chart (from 24 single cells per-population). Data are from one donor, representative of 3. (**e**) Vδ1^+^ T cells were segregated into CD27^hi^ and CD27^lo/neg^, informed by TCR sequence clonality in **d**, and this FACS-gating strategy was applied to focused adults (top row, *n*=13), unfocused diverse adults (second row, *n*=7), and cord blood (third row, *n*=4). Flow cytometry data is shown for representative donors, with all donors shown with mean ±s.e.m. (right column). (**f**) Comparison of Vδ1^+^ CD27^lo/neg^ cells from all adult and cord blood donors (left panel), correlation with CDR3δ D75 (middle) and Chao1 TCRδ1 diversity metric, normalized to 50,000 randomly chosen CDR3 sequencing reads, (right) for each donor. (**g**) TCRδ repertoire analysis of sorted CD27^hi^ and CD27^lo/neg^ Vδ1^+^ T cells, showing Tree maps (top row), accumulated frequency graphs of the top 10 clonotypes (bottom row) and CDR3δ D75 values (right column). (**h**) Comparison of CD27, CCR7, CD62L, CD28 and IL-7Rα expression within CD8^+^ T _EMRA_, CD8^+^ T _naïve_, Vδ1^+^ CD27^hi^ and Vδ1^+^ CD27^lo/neg^ T cells. Histograms from one representative donor and graphs show mean±s.e.m. from 14 different donors. Data analysed by Kruskal–Wallis ANOVA with Dunn's post-test comparisons, *P<0.05, **P<0.01 and ****P<0.0001.

To confirm that the prevalent clonotypes we detected genuinely reflected differential lymphocyte expansion rather than disproportionate expression levels of specific TCR transcripts, we single cell sorted the Vγ9Vδ1 T cell population, which frequently featured highly dominant clonotypes (Fig. 2), from six healthy adult donors, and used RT-PCR to identify Vγ9Vδ1 TCR pairs (Fig. 5a). In each case, the dominant Vγ9 and Vδ1 TCR chain evident from NGS analyses was identified in >50% of sequences (Fig. 5b), confirming that the prevalent clonotypes detected in the NGS analyses truly reflected clonal dominance at the cellular level.

To assess whether the dominant Vδ1 expansions are stably maintained over time, we compared our ARM-based repertoire analyses with an analogous, RNA-based RACE TCR repertoire analysis, conducted previously on 5 individuals from our cohort (2 CMV-seronegative, 3 CMV-seropositive). In 5/5 donors analysed, the most frequent clonotypes from RACE analysis were detected in subsequent ARM analyses, conducted between 12–18 months later (Fig. 5c). Also, in most donors, the hierarchy of prevalent clonotypes was broadly conserved in both analyses (Fig. 5c). Therefore clonotypic expansions prevalent in the Vδ1 T-cell repertoire can be highly stable over time.

Next, we assessed how Vδ1^+^ T-cell differentiation status correlated with the extent of clonotypic focusing. We sorted sub-populations of Vδ1^+^ cells based on expression of CD27 and CD45RA, markers that have been used to identify naïve and memory subsets in CD8 cells^27^ and γδ T cells^16^. Strikingly we observed the vast majority of clonal populations resided in CD27^lo/neg-^ CD45RA^+^ populations (gates C and D), whereas clonotypes found in CD27^hi^ populations (gates A and B) were strikingly diverse (Fig. 5d). Notably, when applying a CD27^hi^ and CD27^lo/neg^ gating strategy to individuals with focused Vδ1 TCR repertoires, they displayed a dominant CD27^lo/neg^ CD45RA^+^ phenotype but also retained a minor CD27^hi^ population, whereas Vδ1 T cells of both cord blood, and Vδ1 diverse adults, were predominantly CD27^hi^ (Fig. 5e). Furthermore, focused donors had elevated percentages of Vδ1^+^/CD27^lo/neg^ cells compared with diverse and cord blood donors (Fig. 5f, left). Moreover, the percentage of CD27^lo/neg^ cells each donor displayed inversely correlated with the TCRδ D75 and Chao 1 indices of Vδ1 diversity (Fig. 5f, right panels). These correlations suggested that Vδ1 TCR repertoire focusing was accompanied by transition of Vδ1^+^ T cells from a CD27^hi^ CD45RA^+^ to a CD27^lo/neg^ CD45RA^+^ phenotype. To test this directly, we sorted Vδ1^+^ CD27^hi^ versus CD27^lo/neg^ populations from two focused adult donors and performed NGS TCR repertoire analysis. This unequivocally confirmed that the Vδ1^+^/CD27^hi^ population displayed a diverse TCRγδ repertoire, generating TCRδ repertoires and TCRδ D75 values similar to cord blood and diverse adult donors, whereas the Vδ1^+^/CD27^lo/neg^ population was comprised of even more pronounced dominant TCR clonotypes (Fig. 5g), compared with each donor's original TCRδ repertoire profile (Fig. 2b). Inter-individual comparisons revealed the majority of Vδ1^+^CD27^hi^ T cells expressed other markers typical of naïve CD8 T cell populations including IL-7Rα, CD28, and CCR7 and CD62L, while Vδ1^+^/CD27^lo/neg^ cells had downregulated surface expression of these markers similarly to memory CD8 compartments (Fig. 5h) (reviewed in Akbar and Fletcher, 2005)^17^. Although CD45RA and CD27 have been used before to assign γδ memory compartments^16^,^21^, our CD27^hi^ *versus* CD27^lo/neg^ gating strategy represents a new approach to immunophenotyping Vδ1 populations, which more closely aligns naïve/effector marker expression to Vδ1 TCR diversity/clonality.

### Vδ1^+^CD27^lo/neg^ clones respond to IL-15 and TCR stimulation

**Figure 6:**
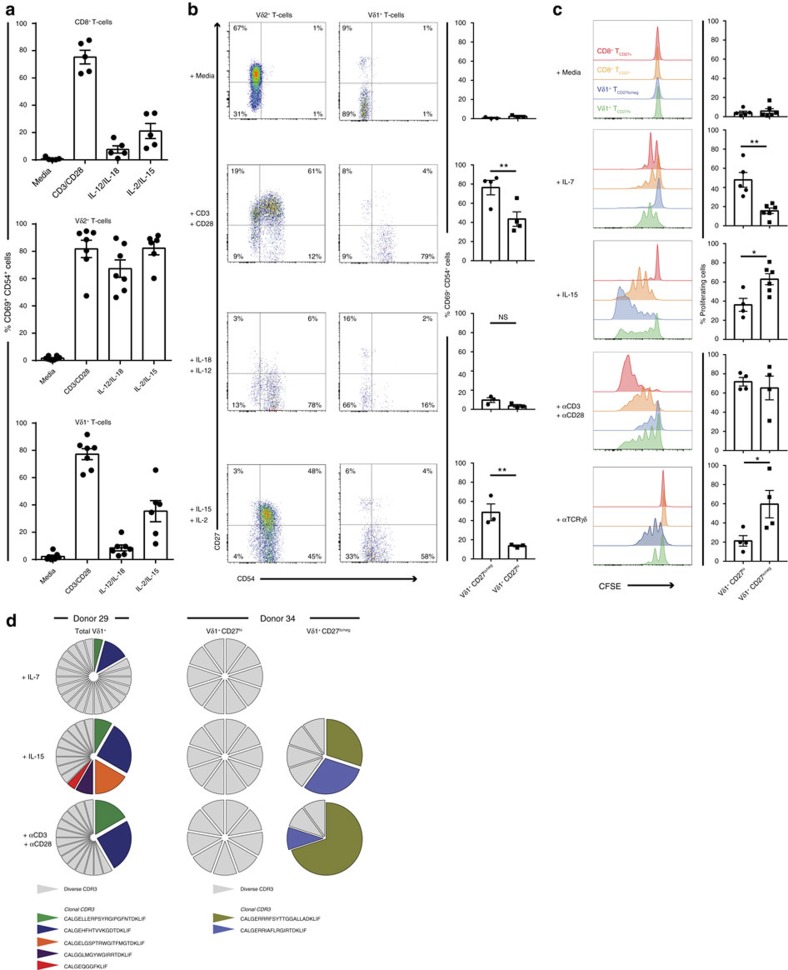
Functional characteristics of clonally expanded CD27^lo/neg^versus naive CD27^hi^ Vδ1^+^ T cells. (**a**) Sorted CD3^+^ T cells were incubated for 72 h with cytokines or anti-CD3/CD28 beads. CD8^+^, Vδ2^+^ and Vδ1^+^ T cells were then assessed for the upregulation of CD69 and CD54. Graphs show mean ±s.e.m. from medium controls (*n*=5–7), CD3/CD28 (*n*=5–7), IL-12/IL-18 (*n*=5–7) and IL-2/IL-15 (*n*=5–6) stimulation. (**b**) Flow cytometry analysis of Vδ2 and Vδ1 T cells from **a**, with cells analysed for CD27 and CD54 expression (left, representative flow cytometry plots) and graphs show mean±s.e.m. of CD69^+^ CD54^+^ cells in Vδ1^+^ CD27^hi^ and Vδ1^+^ CD27^lo/neg^ populations, data from medium controls (*n*=4), CD3/CD28 (*n*=4), IL-12/IL-18 (*n*=3) and IL-2/IL-15 (*n*=3). (**c**) Proliferation of Vδ1 T cells, assessed by CFSE dilution, for 7 days in response to stimulation with IL-7, IL-15, anti-CD3/CD28 and anti-TCRγδ mAb. Histograms are from a representative focused adult donor's Vδ1^+^ CD27^hi^ cells, Vδ1^+^ CD27^lo/neg^ cells, CD8^+^CD27^+^ or CD8^+^ CD27^neg^ T cells and graphs show mean±s.e.m. of proliferating cells (*n*=4–6). (**d**) Total proliferating Vδ1^+^ T cells (Left, donor 29) or CD27^hi^ and CD27^lo/neg^ Vδ1^+^ T cells were single cell sorted and CDR3δ sequenced by PCR, prevalent clonotypes detected by previous deep sequencing are coloured and diverse individual sequences are grey. Donor 29 had 24 single cells, and donor 34 had 10 single cells analysed from each condition. Data analysed by student's *t* test, NS=*P*>0.05, *P<0.05 and **P<0.01.

Comparisons of the functional responsiveness of total Vδ1 T-cell subsets indicated robust CD3/CD28-dependent activation equivalent to CD8^+^ T cells and Vδ2^+^ γδ T cells (Fig. 6a). However, Vδ1^+^ CD27^lo/neg^ T cells displayed a markedly enhanced responsiveness to both CD3/CD28 and IL-15 relative to CD27^hi^ Vδ1 T cells after short-term stimulation (Fig. 6b). Importantly, and unlike Vδ2^+^ T cells, Vδ1^+^ T cells were largely unresponsive to the innate T cell trophic IL-18 or IL-12 cytokines (Fig. 6a,b). In keeping with their high IL-7Rα expression (Fig. 5g), CD27^hi^ Vδ1 subsets proliferated preferentially in response to IL-7 stimulation relative to CD27^lo/neg^ Vδ1 populations, whereas the latter displayed more pronounced IL-15-induced proliferation (Fig. 6c). Moreover, single cell PCR confirmed that cells that proliferated (that is, became CFSE^lo^) in 7-day IL-7 cultures predominantly expressed diverse TCRs, irrespective of whether Vδ1 T cells were analysed on bulk or segregated into Vδ1 CD27^hi^ and Vδ1 CD27^lo/neg^ subsets, whereas expanded clonotypes proliferated preferentially in response to IL-15 (Fig. 6d). In addition, both CD27^hi^ and CD27^lo/neg^ populations responded to CD3/CD28 (Fig. 6c) and, separately, to anti-γδ TCR antibody stimulation in 7-day cultures. CD27^lo/neg^ populations in general exhibited somewhat enhanced proliferative responses to anti-γδ TCR antibody (Fig 6c; *P*=0.0458) relative to CD27^hi^ cells. This suggests that although expanded Vδ1 clones have a CD45RA^+^/CD27^lo/neg^ phenotype normally associated with CD8 T_EMRA_ cells, they are capable of proliferating in response to signals through the TCR and IL-15.

### Vδ1^+^CD27^lo/neg^ clones express cytotoxic markers and CX_3_CR1

**Figure 7:**
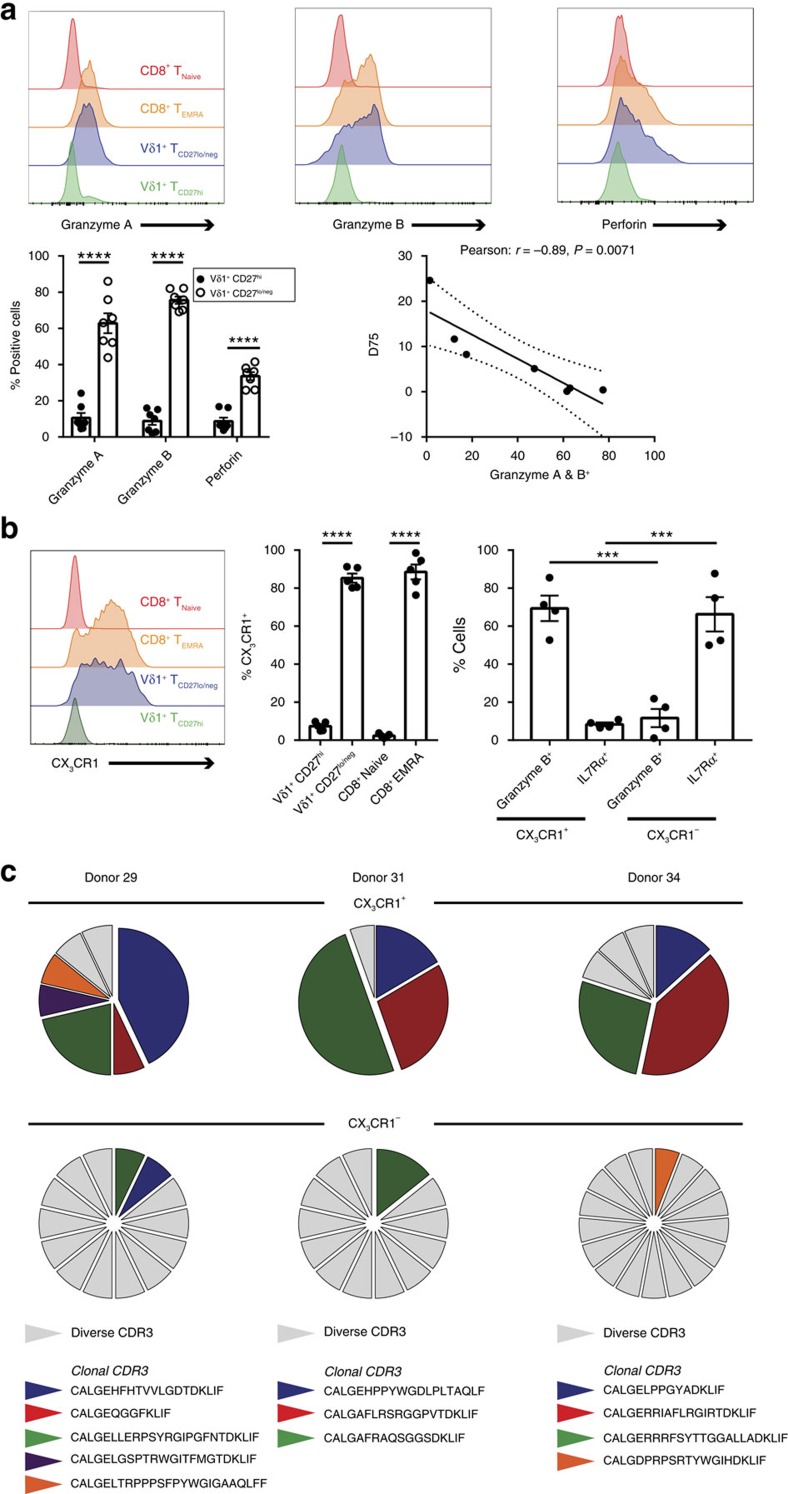
Clonally expanded CD27^lo/neg^Vδ1^+^ T cells express CX_3_CR1 and cytotoxic effector molecules. (**a**) Expression of intracellular cytotoxic effector molecules, granzyme A, granzyme B and perforin by CD8^+^ T _Naïve_, CD8^+^ T _EMRA_, Vδ1^+^ CD27^lo/neg^ and Vδ1^+^ CD27^hi^ cells. Correlation graph between all 7 donors and the corresponding TCRδ D75 calculated from TCR repertoire deep sequencing. Representative histograms, graph and dot plot are from 7 donors. (**b**) Expression of CX_3_CR1 on the surface of CD8^+^ T _Naïve_, CD8^+^ T _EMRA_, Vδ1^+^ CD27^lo/neg^ and Vδ1^+^ CD27^hi^ cells. Within CX_3_CR1^+^ or CX_3_CR1- Vδ1^+^ T cells the expression of granzyme B and IL7Rα. Representative histograms of 5 donors (left), with all 5 donors shown (middle) and 4 of these donors assessed for CX_3_CR1/granzyme B/IL7Rα (right). (**c**) Single cell γδ TCR analysis from 3 donors, single cells were sorted from CX_3_CR1^+^ or CX_3_CR1- Vδ1^+^ T cells and CDR3 sequences analysed against dominant clonotypes identified by deep sequencing. Graphs show the mean±s.e.m. and data were analysed by two-way ANOVA (**a**) and one-way ANOVA with Holm-Sidak's post-tests (**b**), ***P<0.001 and ****P<0.0001.

A hallmark of effector T cell subsets is the acquisition of intracellular cytotoxic granules, therefore we assessed intracellular cytotoxic marker expression in both CD27^hi^ and CD27^lo/neg^ Vδ1 T-cell subsets (Fig. 7a). CD27^hi^ Vδ1 T cells largely lacked Granzyme A or B and perforin expression, whereas these markers were markedly upregulated in CD27^lo/neg^ Vδ1 T cells, again matching levels in naïve and effector memory CD8^+^ T-cell subsets, respectively. Next, we assessed the proportion of Vδ1 T cells that expressed Granzyme A/B and found this significantly correlated with the extent of focusing (TCRδ D75, *P*=0.0071). CX_3_CR1 (fractalkine receptor) was preferentially expressed in CD27^lo/neg^ versus CD27^hi^ naive-like Vδ1^+^ T cells (Fig. 7b) and CX_3_CR1 expression in Vδ1^+^ T cells exclusively marked out Granzyme B^+^ cells whereas CX_3_CR1^neg^ Vδ1^+^ T cells predominantly expressed IL-7Rα (Fig. 7b). We then assessed TCR usage on the single cell level and in each donor, CX3CR1^+^ cells within the Vδ1^+^ T-cell population were composed exclusively of clonotypically expanded sequences, with each sequence identity matching those detected in deep sequencing analysis, whereas CX3CR1^neg^ cells were predominantly composed of multiple diverse TCRs (Fig. 7c). Together, these data indicate that clonal expansion is inextricably linked to changes in homing receptor, CD27 and cytolytic effector expression.

## Discussion

γδ T cells have been highlighted as a key cellular exemplar of lymphoid stress surveillance, which invokes unconventional lymphocytes that can quickly initiate responses to stress challenges without the obligatory delay associated with clonal expansion and differentiation^28^, a paradigm thought to apply to some murine γδ TCR subsets^29^. Although Vδ2^+^ cells increase in number upon microbial challenge^30^, they arguably fit this paradigm^7^. However, how it applies to human Vδ2^neg^ T cells has to date remained unclear.

Our findings expose several features of Vδ1 T cells, which comprise the dominant proportion of Vδ2^neg^ T cells, that differ fundamentally from this paradigm. Instead of being pre-expanded, in most adults a small number of specific clonotypes emerge from an initially unfocused neonatal Vδ1 T-cell repertoire, undergo pronounced clonal expansion, and ultimately dominate the Vδ1 γδ T-cell compartment. Notably, such clonotypic expansions were relatively stable over time, and their occurrence was not an inevitable consequence of a maturing γδ T-cell compartment, as a sizeable minority of adults had largely unfocused repertoires. CDR3 sequences of dominant clonotypes were extremely complex, suggesting heavy selection of initially low frequency clonotypes resulting from single, unlikely recombination events. Consistent with this, Vδ1 TCR sequences observed were, overwhelmingly, unique to each individual, indicating the Vδ2^neg^ repertoire, including dominantly expanded clonotypes, is essentially private. Conversely, analyses highlighted Vγ9/Vδ2 cells shared sequences in TCRγ and (to a lesser extent) TCRδ, confirming this subset featured a semi-invariant TCR.

Importantly Vδ1 clonal expansion was accompanied by phenotypic differentiation and a distinct functional biology. Unfocused adult TCR repertoires were associated with apparently naïve Vδ1 populations characterized by a CD27^hi^CCR7^+^CD28^+^IL-7Rα^+^CD62L^+^ phenotype evident in all individuals but predominant in the diverse adult subgroup; moreover, cord blood Vδ1 cells largely conformed to this phenotype. Conversely, clonally expanded populations were analogous to CD8 T effectors in displaying a CD27^lo/neg^CCR7^neg^CD62L^lo^CD28^neg^IL-7Rα^neg^ phenotype, and similarly were marked out by CX_3_CR1 expression. Relative to CD27^hi^ Vδ1^+^ cells, CD27^lo/neg^ Vδ1 subsets displayed more rapid activation and proliferation in response to CD3/TCR stimulation, upregulation of multiple cytotoxic markers equivalent to CD8 EMRA subsets, differential cytokine responsiveness (enhanced to IL-15 and a lack of response to IL-7), and differential homing receptor expression, consistent with a functional effector status. In contrast, CD27^hi^ Vδ1^+^ T cells were responsive to IL-7, expressed secondary lymphoid homing receptors, displayed relatively slow proliferation kinetics after TCR stimulation, and largely lacked expression of perforin/granzymes, consistent with a functionally naïve status. Collectively, these findings reveal a fundamentally adaptive biology for Vδ1 T cells, and suggest the γδ TCR has a central role in driving this biology. While they do not formally exclude the possibility that stochastic processes could explain the expansions we observe, they strongly support a model involving clonal selection of naïve Vδ1 T cells that express functionally useful TCRs enabling responses to microbial/non-microbial stress challenges encountered, accompanied by differentiation to an effector phenotype. They argue against the idea that Vδ1 clonotypic focusing merely represents an immunological imprint of past stress challenges, and instead are more suggestive of long-lived, highly specific, functional γδ T-cell memory that enables augmented responses to recurrent stress challenges, akin to classical immunological memory, although importantly, not MHC-restricted.

The model outlined above shares several key tenets with classical adaptive immunity, but differs critically in being MHC-unrestricted, and represents an unconventional mode of adaptive immune surveillance. Although one limitation of our study is that it focuses predominantly on Vδ1 T cells, our repertoire data highlight that Vδ3 and Vδ8 populations exhibited a degree of clonotypic focusing and were equivalently private to Vδ1 repertoires, suggesting the biology we observe for Vδ1 T cells could also apply to other human Vδ2^neg^ T cells. However, in several key respects this model does not apply to Vγ9/Vδ2 T cells. In contrast to Vδ1 populations our Vγ9/Vδ2 TCR repertoire data confirm highly restricted CDR3 lengths, including prevalent Vγ9 sequences of limited complexity that were common to multiple individuals. Consistent with these data, Dimova *et al*.^7^ detected prevalent Vγ9 sequences that were present at birth in multiple individuals, irrespective of pathogen exposure. These observations are consistent with a semi-invariant, innate-like biology for the Vγ9/Vδ2 subset that is in keeping with their polyclonal activation by pyrophosphate antigens. In contrast, a confluence of features we observe in Vδ1 T cells, which include profound clonotypic expansions of often highly complex TCRs from an initially completely unfocused, private repertoire, and concomitant phenotypic differentiation involving loss of secondary lymphoid homing markers and upregulation of effector molecules, point to a very different adaptive biology.

This model raises a number of questions, including regarding Vδ1–positive T-cell homing. The majority of naïve CD27^hi^ Vδ1 subsets expressed both CCR7 and CD62L, suggesting they may access secondary or alternatively tertiary lymphoid organs, and may become primed there before differentiating to CD27^lo/neg^ effectors, which are CCR7^neg^ CD62L^lo^, but also display CX3CR1 upregulation, which may affect peripheral or endothelial homing. How this transition occurs is unclear, however conceivably dendritic cells, which prime αβ T-cell responses, may also prime MHC-independent Vδ1 responses. How our results relate to Vδ2^neg^ development in solid tissues, where they represent the dominant γδ T-cell subset, is unclear, but could potentially involve central or local priming events. Also, the kinetics of Vδ2^neg^ T-cell responses merit further investigation, but might parallel those of conventional αβ T-cell adaptive responses.

Although the stress challenges underlying Vδ2^neg^ clonotypic focusing are uncertain, one obvious possibility is infection. Although Vδ2^neg^ T-cell responses have been strongly linked with CMV infection^11^,^15^ they are not limited to this setting, since we found CMV-seronegative individuals also exhibited extreme clonotypic focusing. Further studies assessing in parallel Vδ2^neg^ TCR repertoire focusing and microbial exposure, including during stress stimuli such as acute CMV infection, will shed light on how Vδ2^neg^ adaptive immune surveillance operates during relevant infectious challenges. Our model would predict a small number of previously low frequency clonotypes may become heavily expanded during/after such challenges. Moreover, dissecting the reasons why some adults retain largely unfocused, naive Vδ1 repertoires is important. Although differential pathogen exposure may be one factor, ‘holes' might conceivably exist in the Vδ1 TCR repertoires of certain individuals, resulting in an inability to respond to a specific pathogen. In addition, while we introduced a binary delineation of our cohort into ‘focused' and ‘diverse' individuals, both CD27^hi^ and CD27^lo/neg^ Vδ1 subpopulations were present in all individuals, highlighting both the universal nature of the adaptive biology we observe and also the fact that in reality there is likely to be a continuous spectrum of diversity. Understanding when clonotypic focusing occurs in life is clearly important, including in neonates, when conventional adaptive immunity is less potent and γδ T-cell responses may play an important role^31^,^32^. Further studies are required to more comprehrensively explore Vδ1 repertoire focusing throughout life, and how this links with pathogen exposure.

How Vδ2^neg^ adaptive immune surveillance relates to γδ TCR ligand recognition merits consideration. As the focusing we observe is highly CDR3-specific, even within the same chain pairing combination, we hypothesize it results from TCR ligand-dependent selection. The identity of ligands for such specificities is largely unclear (see below), but might include self-encoded and/or foreign proteins^3^,^33^,^34^,^35^,^36^,^37^. Furthermore, although the private TCR repertoires we observe may reflect reactivities restricted to each individual, they do not formally exclude the possibility of degenerate recognition of conserved ligands by diverse TCRs.

Studies on Vδ2^neg^ T cells have highlighted a diverse and somewhat confusing range of TCR ligands. While these ligands highlight the broad recognition capabilities of the Vδ2^neg^ γδ TCR, their physiological importance is less clear. For future studies, our model strongly suggests that natural *in vivo* expansion and phenotypic differentiation may be important considerations in assessing the biological relevance of proposed γδ TCR ligands to adaptive immune surveillance by Vδ2^neg^ T cells, and for prioritising clonotypes for ligand identification. From this perspective, while unequivocal binding and/or structural data have been determined for phycoerythrin (PE) - and CD1d-reactive Vδ2^neg^ TCRs^33^,^35^,^37^, both PE and CD1d reagents published to date stain a small percentage (<0.1%) of Vδ1 T cells in peripheral blood in the absence of *in vitro* expansion, and the naïve/effector phenotype of such cells is unclear. While such cells clearly do not represent dominantly expanded blood clonotypes, reactive populations could be preferentially localized to solid tissues or be lipid-specific. By analogy, notably CD1d-restricted iNKTs comprise a minor component of αβ T cells in peripheral blood but are highly prevalent in liver^38^. Furthermore, conceivably expanded clonotypes could recognize particular CD1d-presented lipids. Further studies are required to resolve these questions.

In this context, γδ TCR specificities present on Vδ2^neg^ T cells naturally expanded during infection, which include the LES^36^ and POS4 (ref. 39) TCRs, may be particularly relevant. The LES TCR, which directly binds Endothelial Protein C Receptor^36^, conceivably represents a molecular exemplar of Vδ2^neg^ adaptive immune surveillance. Naturally expanded during acute CMV infection to∼20% of total T cells, the LES clone was CD28^neg^CD45RO^neg^, suggestive of an effector phenotype^40^. Also, the LES TCR utilizes a highly complex TCRδ rearrangement (incorporating 14 N/P additions) and a simpler TCRγ chain, typical of many of the Vδ2^neg^ dominant clonotypes we observed in this study. However LES TCRδ exact sequences were not observed in any individual from our cohort, and at least the CDR3 region of the LES TCRγ chain has been highlighted as important for LES/EPCR ligand binding^36^. These observations not only indicate the LES TCR was private to the individual in which it originated, but, raise the possibility that reactivity to EPCR might also be private, consistent with failure of previous attempts to identify reactivity to EPCR in other individuals^36^.

The Vγ8Vδ1 POS4 TCR, also derived from a clonotype expanded in acute foetal CMV infection^39^, would superficially appear to conflict with this study, as it was observed in multiple individuals. Tellingly, POS4 is exceptional in comprising CDR3γ and CDR3δ both resulting from recombination of germline-encoded segments without any N/P nt addition (Supplementary Tables 2 and 3). We suggest this TCR is preferentially generated in gestation before TdT is expressed at 20 weeks^41^, and that its expansion during acute infection may reflect recognition of an important CMV-associated (although not necessarily CMV-encoded) ligand. Therefore, while our data stress the overwhelmingly private nature of the Vδ2^neg^ TCR repertoire, the POS4 TCR clonotype, because of its generation and expansion *in utero*, may be the ‘exception that proves the rule', relative to most adult peripheral TCR sequences.

Pre-expanded, semi-invariant T cells such as MAIT, iNKT and some γδ T-cell subsets, as well as NK cells, may permit rapid responses to stress challenges such as pathogen infection, before conventional adaptive immunity has time to develop^28^. What advantage would a parallel Vδ2^neg^ adaptive immune surveillance system provide, involving clonal selection of private TCR clonotypes from a diverse repertoire? Possibilities include the potential for amplification of the stress response, and immunological memory to recurrent stress stimuli, both justifying further investigation. Secondly, the system may permit diverse routes to recognition of ‘altered self'. One potential paradigm was suggested by LES reactivity to EPCR, which enabled functional reactivity to CMV-infected endothelial cells (a key target for CMV infection *in vivo*) *via* a TCR-extrinsic, CMV-induced ‘multimolecular stress signature'^36^. However additional paradigms will no doubt emerge from identification of further ligands for clonotypes naturally expanded *in vivo*. Finally, another important consideration is the protection from pathogen immune escape mechanisms provided by Vδ2^neg^ adaptive immune surveillance versus stress sensing by alternative routes such as innate immune subsets, NK cells, and semi-invariant T cells. Generally, these alternative cell types rely on conserved germline-encoded receptors/ligands such as PRRs that sense PAMPs/DAMPs, NKG2D that recognizes MHC-like stress antigens, and MHC-like molecules such as MR1/CD1. Notably, there is strong evidence for pathogen escape from both PRRs^42^ and NKG2D^43^. Morever, CD1 molecules recognized by NKT cells, like class I MHC molecules themselves, are targeted by various viral proteins^44^, and MAIT cells are decreased in HIV^45^. In contrast, the evolution of a parallel adaptive immune surveillance system, involving a radically different biology centred on clonal amplification from within a diverse, private Vδ2^neg^ antigen receptor repertoire, and potentially exploiting diverse ligands private to each individual, may provide a much greater challenge for pathogens to evade.

Vδ2^neg^ T cells are strongly implicated in pathogen-specific^11^ and anti-tumour immunity^46^, and are the subject of increasing immunotherapeutic interest. Three aspects of the adaptive biology we outline here have translational relevance. Firstly, major differences in the ability of specific, private Vδ2^neg^ γδ TCRs to mediate physiologically relevant antigen recognition events would open the possibility of identifying and exploiting optimal Vδ2^neg^ TCR clonotypes (for example, *via* T-cell engineering), to enhance pathogen/cancer-specific immunity. Secondly, the longevity of dominant clonotypes suggests interaction of vaccination approaches with Vδ2^neg^ immune surveillance merits further investigation, and might be manipulable to enhance vaccine protection. Finally, a greater understanding of the relationship between different Vδ2^neg^ TCR ligands, their mode of recognition, tissue expression and regulation/dysregulation, will undoubtedly provide novel therapeutic avenues and insights.

*Note added in proof:* Consistent with the suggestion that viral infection may be one stress challenge that drives clonal expansions in the Vδ2-negative compartment, a recent study from Ravens *et al*. and published in Nature Immunology (doi: 10.1038/ni.3686) has highlighted clonotypic γδ T cell expansions following CMV reactivation after allogeneic stem cell transplantation. 

## Methods

### T cell isolation and culture and activation

Human peripheral blood mononuclear cells (PBMC) were isolated from heparinized venous blood from consenting healthy donors (protocol approved by the NRES Committee West Midlands ethical board; REC reference 14/WM/1254). Briefly, blood was layered over lymphoprep (Stem Cell Technologies) with resulting PBMC used for subsequent experiments. Plasma was also harvested for CMV IgG (ELISA kit) measurement. For repertoire analysis, all T-cell populations were sorted directly into RNA*later* (Sigma) or RLT buffer (Qiagen) supplemented with β-mercaptoethanol (Sigma) on a MoFlo Astrios Cell Sorter (Beckman Coulter). For activation and proliferation of T cells, either PBMC or isolated CD3^+^ T cells, obtained by positive magnetic bead isolation (Miltenyi), were labelled or not with 0.3 μM CFSE (eBioscience) and cultured with cytokines, 25 ng ml^−1^ IL-7 (Peprotech), 100 IU ml^−1^ IL-2, 25 ng ml^−1^ IL-15, 5 ng ml^−1^ IL-12, 5 ng ml^−1^ IL-18 (all Miltenyi), and/or antibodies directed against CD3 (OKT3; eBioscience), CD28 (28.2), mIgG1κ (MOPC-21), TCR γδ (B1; all Biolegend) or CD3/CD28 T activator beads (Invitrogen), where indicated and for up to 7 days in RPMI-1640 medium (Invitrogen) supplemented with 2 mM L-glutamine, 1% sodium pyruvate, 50 μg ml^−1^ penicillin/streptomycin (Invitrogen) and 10% fetal calf serum (Sigma).

### Antibodies and flow cytometry

For cell sorting, Vδ1^+^ and Vδ2^+^populations were labelled with anti-CD3 (UCHT1; 1:100), TCR αβ (IP26; 1:50), TCR Vδ1 (TS8.2 or REA173; 1:100) or TCR Vδ2 (B6; 1:100) and where indicated CD27 (M-T271; 1:200) and CD45RA (HI100; 1:200). Alternatively, Vδ2^neg^ T cell populations were sorted by labelling with anti-CD3 (UCHT1; 1:100), TCR αβ (IP26; 1:50), TCR γδ (IMMU510; 1:200), TCR Vδ2 (B6; 1:100), although this sorting strategy was subject to antibody interference leading to Vδ2^+^ sequences contaminating TCR repertoire analyses. For phenotypic analysis, freshly isolated, frozen PBMC or cultured cells were labelled with Zombie Aqua viability dye (Biolegend), and the cells were then subsequently stained for cell surface antigens with antibodies directed against CD3 (UCHT1 or HIT3a; 1:100), CD8 (SK1; 1:200), CD45RA (HI100; 1:200), CD27 (M-T271; 1:200), CCR7 (G043H7; 1:100), CD62L (DREG-56; 1:100), CD28 (28.2; 1:80), CD161 (HP-3G10; 1:100), CD16 (3G8; 1:100), CD69 (FN50; 1:100), CD25 (2A3; 1:100), CD54 (HA58; 1:100), TCR Vδ2 (B6; 1:100), TCR γδ (B1; 1:100), TCR αβ (IP26; 1:50); all Biolegend. CD127 (IM1980U; 1:20), TCR γδ (IMMU510; 1:200) and TCR Vγ9 (IMMU360; 1:400); Beckman Coulter. TCR Vδ1 (TS8.2; 1:100); Fisher Scientific. TCR Vδ1 (REA173; 1:100) and TCR Vδ2 (123R3; 1:200); Miltenyi. For intracellular staining, after surface antibody staining cells were fixed in IC Fixation buffer (eBioscience) and finally stained in Permeabilisation Buffer (eBioscience) with antibodies directed against Granzyme A (CBO9; 1:100), Granzyme B (GB11; 1:100) and Perforin (B-D48; 1:80); all Biolegend. Cells were acquired on an LSR II (Beckton Dickinson) and data analysed with FlowJo V10.1 (TreeStar).

### RNA-based TCR repertoire analysis

RNA was purified from sorted cells (cell numbers detailed in Supplementary Table 1 and FACS sorting strategy identified in Supplementary Fig. 8) using an RNAmicro kit (Qiagen) according to the manufacturer's instructions. For high throughput deep sequencing of TCRs, we used amplicon rescued multiplex (ARM)-PCR and NGS methods^47^ to analyse all sorted γδ T cell populations. Following initial first-round RT-PCR using high concentrations of gene-specific primers universal primers were used for the exponential phase of amplification^48^ (Patent: WO2009137255A2), allowing deep, quantitative and non-biased amplification of TCR γ and TCR δ sequences. All cDNA synthesis, amplification, NGS library preparation and sequencing were performed by iRepertoire, Inc. (Huntsville, USA). We analysed positively sorted Vδ1^+^ γδ T cells from CMV-seronegative (*n*=10), CMV-seropositive individuals (*n*=10), Umbilical Cord blood (*n*=5; Anthony Nolan Trust, Nottingham), TCR γδ^+^ Vδ2^neg^ T cells (*n*=4) and Vδ2^+^ T cells (*n*=4). For low throughput TCR repertoire sequencing, Vd1^+^ T cells were sorted into RNA*later* using an ARIA II (BD) flow sorter. cDNA was synthesized using SMARTer RACE cDNA Amplification Kit (Clontech) and TCRγ or TCRδ sequences were amplified using a template-switch anchored RT-PCR with a 3′ Cγ or Cδ specific primer. Amplicons were cloned into a TOPO-TA vector (Life Technologies), transformed into *Escherichia coli*, and 96 colonies sequenced.

### TCR sequence analyses

The CDR3 length was defined as the number of amino acids between the second Cys of the V region and the Phe of the J region, according to IMGT. N and P nucleotides were identified using the IMGT Junction Analysis tool^49^,^50^. Vγ9 and Vδ2 sequence logos were generated on the Seq2Logo server^51^ in Shannon format without the use of pseudocounts, and give a visual representation of amino acids enriched at different positions in the observed CDR3 sequences. The different amino acids are coloured according to physicochemical properties (acidic (DE), red; basic (RKH), blue; hydrophobic (ACFILMPVW), black; and neutral (NGSGTY), green). For TCRγ sequences from Vδ2^+^ cells, the 20 most prevalent CDR3γ of 14aa from each of the four adult donors were included (80 sequences in total). For TCRδ sequences, the ten most abundant clonotypes of 13-15 amino acids using Vδ2-Jδ1 from each donor were aligned using Clustal Omega^52^ with default parameters, before logo generation. Narrower bars in the sequence logo correspond to gaps in the sequences.

### Single cell PCR analysis of Vδ1^+^ T cells

PBMC were labelled as above and Vδ1^+^ T cells were single cell sorted directly into individual wells in a 96-well plate containing 2 μl of Superscript VILO cDNA synthesis kit reaction mix (ThermoFisher) containing 0.1% Triton X-100, and incubated according to manufacturer's instructions. TCRγ and TCRδ cDNAs were amplified by two rounds of nested PCR using GoTaq mastermix (Promega) and following primers: for Vδ1, CAAGCCCAGTCATCAGTATCC (external) and CAACTTCCCAGCAAAGAGATG (internal); for Cδ GCAGGATCAAACTCTGTTATCTTC (external) and TCCTTCACCAGACAAGCGAC (internal); for Vγ1-8 ctggtacctacaccaggaggggaagg (external) and TGTGTTGGAATCAGGAVTCAG (internal); for Vγ9 AGAGAGACCTGGTGAAGTCATACA (external) and GGTGGATAGGATACCTGAAACG (internal) and for Cγ CTGACGATACATCTGTGTTCTTTG (external) and AATCGTGTTGCTCTTCTTTTCTT (internal). PCR products were separated on 1.2% agarose gels, and products of successful reactions were incubated with ExoSAP-IT PCR cleanup enzyme (Affymetrix) before sequencing with BigDye Terminator v3.1 (Applied Biosystems) following manufacturer's instructions and cleanup and running on an ABI 3730 capillary sequencer (Functional Genomics Facility, University of Birmingham).

### TCR repertoire data analysis

V, D and J gene usage and CDR3 sequences were identified and assigned and tree maps generated using iRweb tools (iRepertoire, Inc, Huntsville, AL, USA)^53^. Tree maps show each unique CDR3 as a coloured rectangle, the size of each rectangle corresponds to each CDR3s abundance within the repertoire and the positioning is determined by the V region usage. For more detailed analysis and error correction data sets were then processed using the MiXCR software package^54^. Diversity metrics, clonotype overlap and gene usage were plotted in R, by VDJTools^55^. Data are either presented as normalized (each unique CDR3 is assigned a count of one, regardless of frequency), or as non-normalized (which takes into account the frequency of each unique CDR3).

### Statistical analysis

Tabulated data were analysed in Graphpad PRISM 7 (Graphpad Software Inc) and sequencing data by VDJTools^55^. Each data set was assessed for normality using Shapiro-Wilko normality tests. Two-tailed Student's *t*-tests were used for normally distributed data and Mann-Whitney for non-parametric data. Differences between groups were analysed using one-way ANOVA with Holm-sidak's post-tests for normally distributed data or with Kruskal–Wallis ANOVA and Dunn's post-tests for non-parametric data; two-way ANOVA was used when comparing groups with independent variables. *P<0.05, **P<0.01, ***P<0.001 and ****P<0.0001. Correlation was assessed for normally distributed data with Pearson's correlation coefficient or Spearman correlation for non-parametric data.

### Data availability

The sequence data that support the findings of this study have been deposited in the NIH NCBI sequence read archive (SRA) database with the primary accession code SRP096009.

## Additional information

**How to cite this article:** Davey, M. S. *et al*. Clonal selection in the human Vδ1 T cell repertoire indicates γδ TCR-dependent adaptive immune surveillance. *Nat. Commun.* **8**, 14760 doi: 10.1038/ncomms14760 (2017).

**Publisher's note:** Springer Nature remains neutral with regard to jurisdictional claims in published maps and institutional affiliations.

## Supplementary Material

Supplementary Information Supplementary Figures and Supplementary Tables
