# Supplementary material for: Clonal selection in the human Vδ1 T cell repertoire indicates γδ TCR-dependent adaptive immune surveillance
Source: Nat Commun. 2017 Mar 1;8:14760. doi: 10.1038/ncomms14760 (PMC5337994; doi:10.1038/ncomms14760)
Supplement: Supplementary Information — Supplementary Figures and Supplementary Tables [file ncomms14760-s1.pdf]

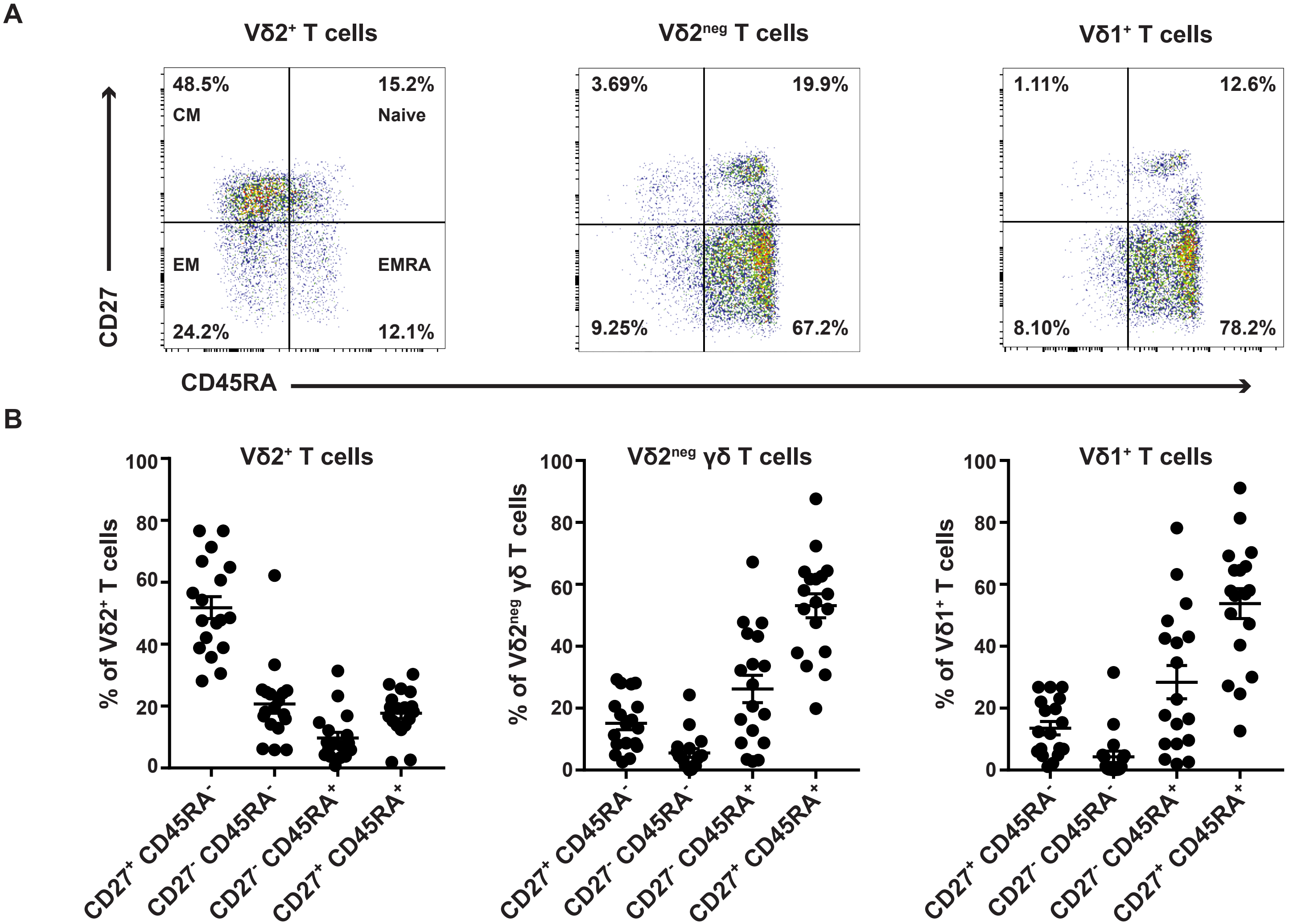

Supplementary Figure 1: Memory phenotype of peripheral blood γδ T cells populations. A and B. Representative flow cytometry plots and graphs show CD27 and CD45RA T cell memory marker expression on gated populations of $CD3^{+}$ $\alpha\beta^{neg}$ γδ T cells: $V\delta2^{+}$, $V\delta2^{neg}$ and $V\delta1^{+}$ T cells. Flow cytometry plots are from one donor and are representative of the graphed data, n=18.

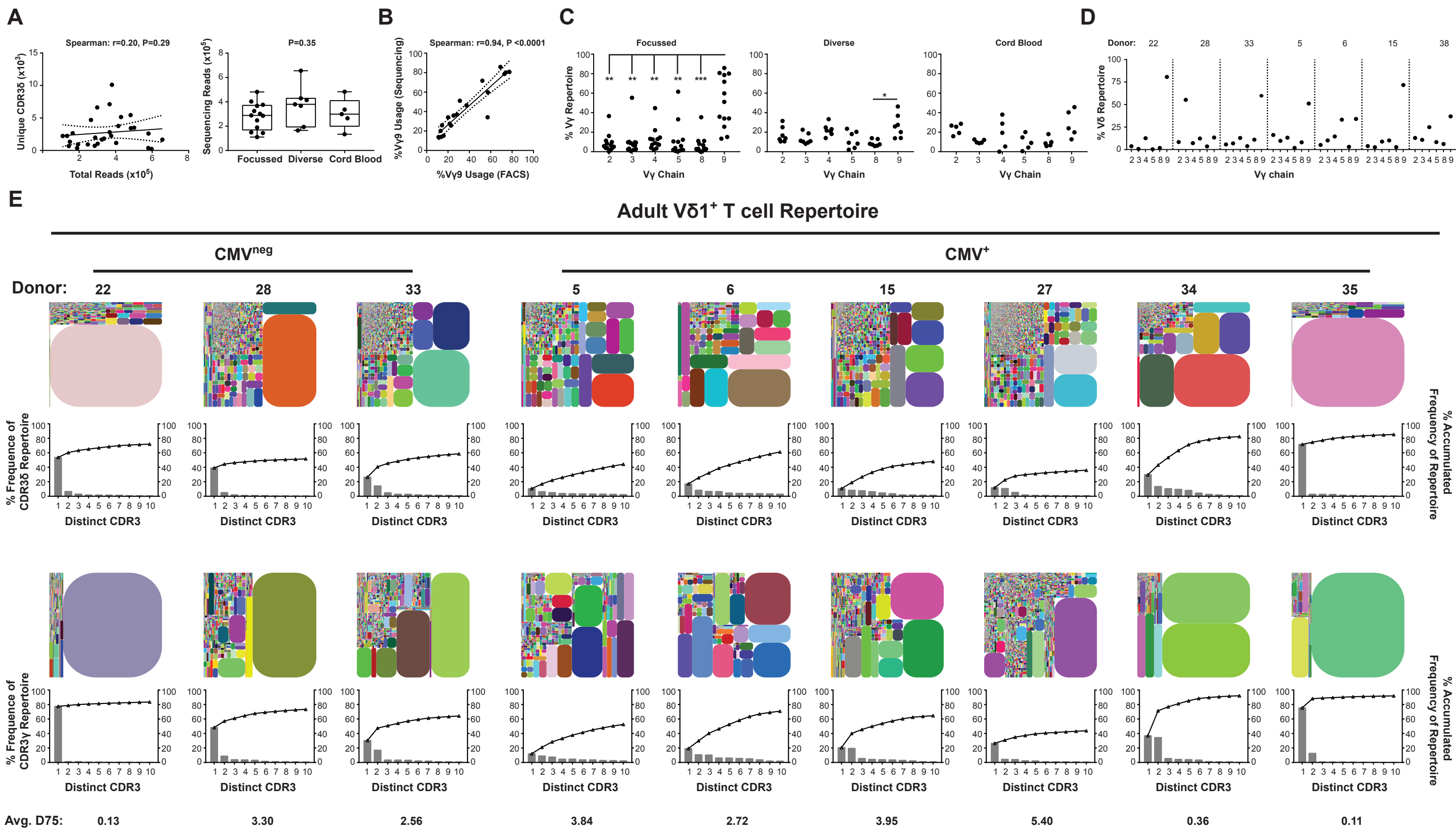

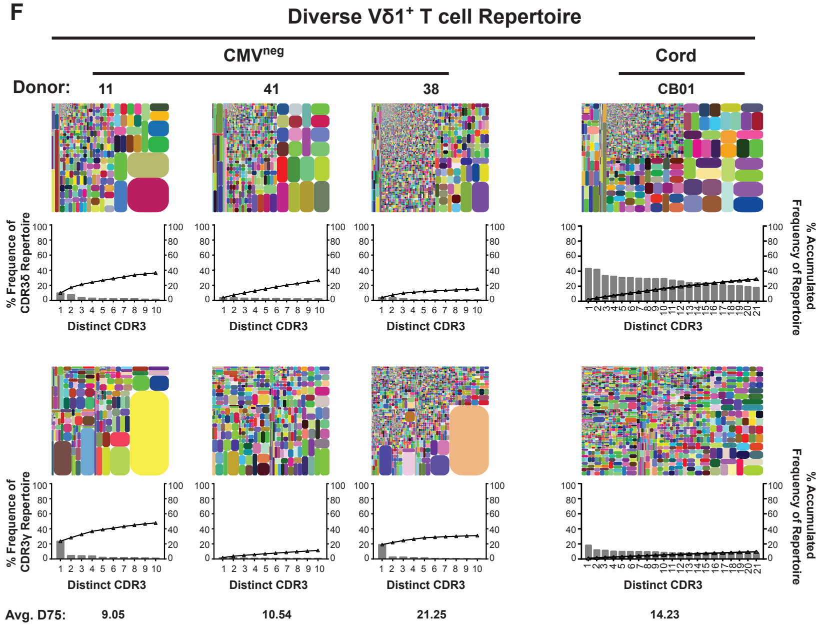

Supplementary Figure 2: Clonotypic focussing in the Vδ1⁺ T cell compartment. A. Comparison of total sequencing reads against the number of unique CDR3s determined for each donor, n=25 (left) and the mean ±SEM of total sequencing reads for each donor grouping (right), focussed (n=13), diverse (n=7) and cord blood (n=5). Data analysed by one-way ANOVA. **B.** Correlation of Vγ9 frequency in Vδ1⁺ T cells obtained by TCR-repertoire sequencing and flow cytometry for each donor (n=20). **C.** Grouped Vγ chain usage in Vδ1+ TCR repertoire sequencing for each donor, focussed (n=13), diverse (n=7), and cord blood (n=5) donors. Data were analysed by Kruskal-Wallis ANOVA with Dunn's post-test comparisons, * $P<0.05$, ** $P<0.01$ and *** $P<0.001$. **D.** Vγ chain usage by Vδ1⁺ T cells from remaining donors not shown in Fig. 2. **E** and **F.** Additional data from adult focussed (**E**), and adult diverse and cord (**F**) donor groupings. These show tree maps of CDR3γ and δ clonotype usage in relation to repertoire size (each CDR3 colour is chosen randomly and does not match between plots) and graphs showing the individual clone frequency (left y-axis) and the accumulated frequency for the first 10 most prevalent clonotypes (right y-axis), for the donors not shown in Fig 2B, E and Fig 3E.

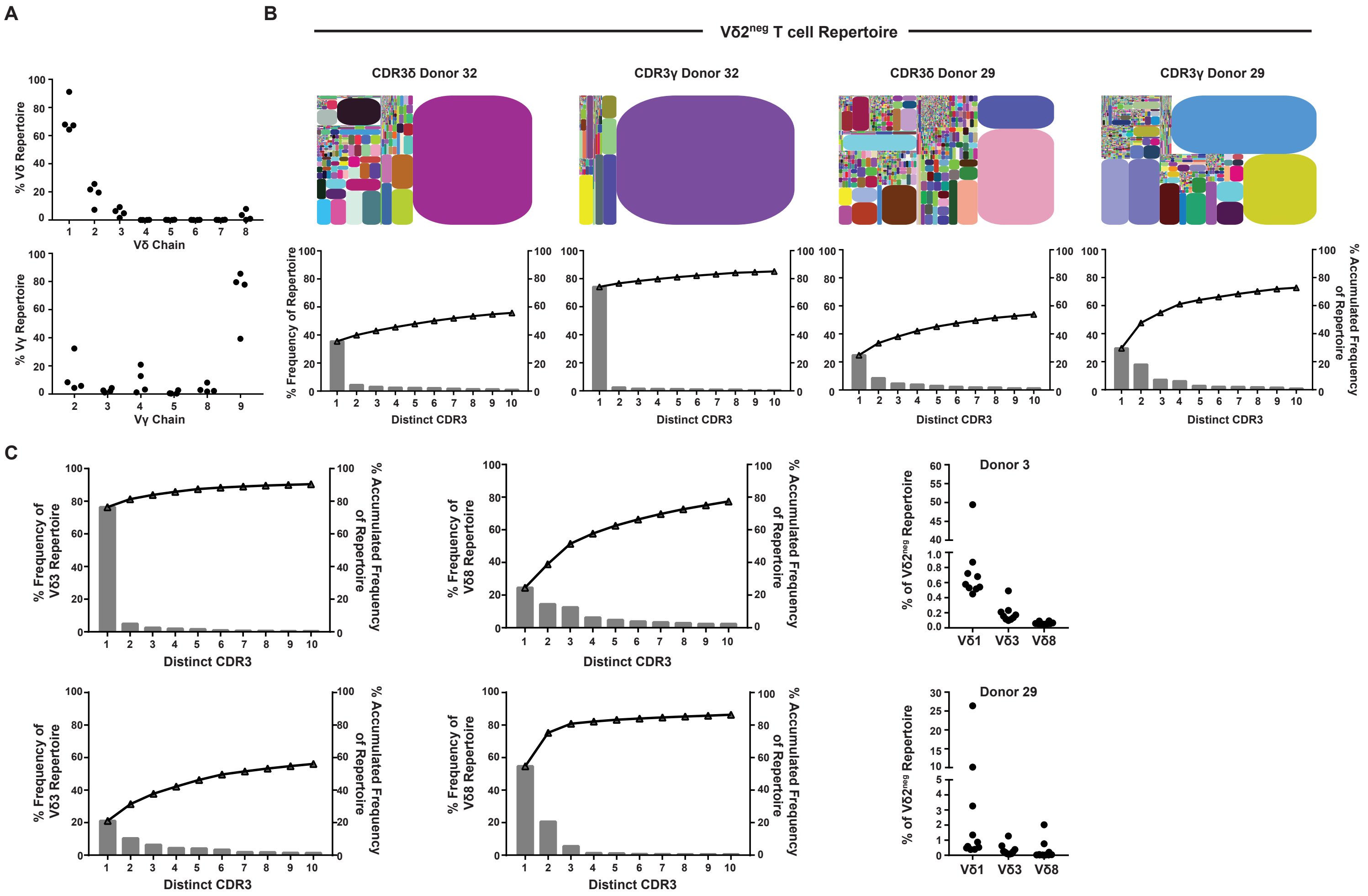

Supplementary Figure 3: γδ TCR repertoire analysis of adult Vδ2^{neg} γδ T cells. A. Vγ and Vδ chain usage in Vδ2^{neg} TCR repertoire data from 4 individuals. **B.** Tree maps showing CDR3 clonotype usage in the Vδ2^{neg} γδ subset in relation to repertoire size and graphs show individual clone frequency against accumulated frequency for the top 10 most prevalent clonotypes. Data are representative of 4 individuals. **C.** Accumulated frequency plots for Vδ3 and Vδ8 clonotypes detected within the Vδ2^{neg} γδ repertoire (left and middle) and a comparison frequency of the top 10 clones in each of Vδ1, Vδ3, Vδ8 filtered repertoires (right). Data are from 2 different donors.

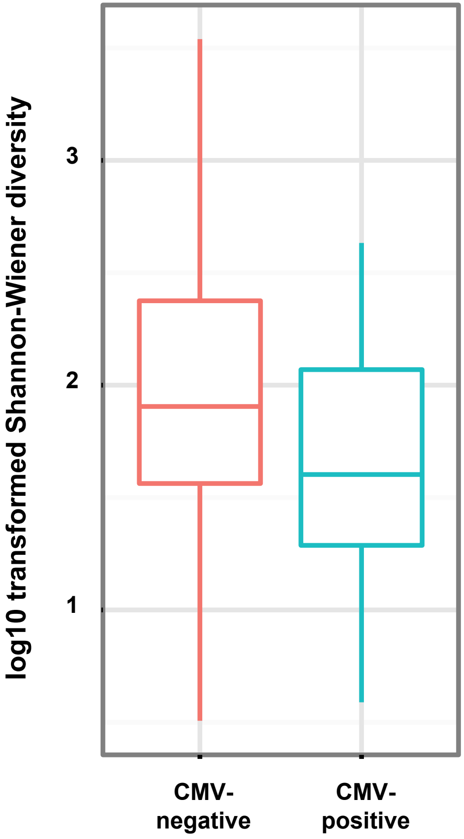

Supplementary Figure 4: CDR3δ1 diversity in CMV⁺ and CMV^{neg} donors. Box plots show mean ±SD of the Shannon-Wiener index of CDR3δ1 diversity in CMV-seronegative (n=10) and CMV-seropositive (n=10) donor TCR repertoires, with differences between groups measured by students t-test, but not significant; p= 0.15.

A

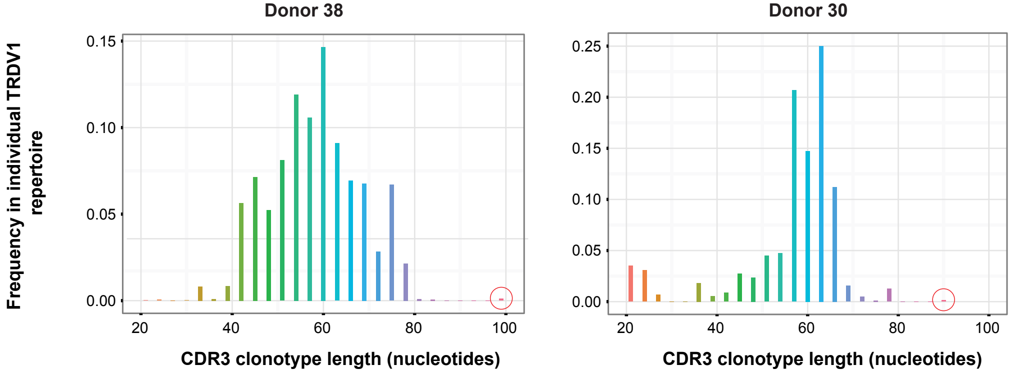

B

IMGT Junction analysis of well-represented extra-long TRDV1-TRDJ1 CDR3 sequences

Donor	Reads, count	Reads, %	CDR3 nucleotide sequence: V-Region N-insertions P-insertions TRDD1 TRDD2 TRDD3 J-Region	Length, nt	N/P nt
6	32	0.048%	TGTGCTCTTGGGGAACGGGTCGCGCTCCGGCGGTCTTCCAAGGATTACTGGGGATACGACGGAGGTACACCGATAAACTCATCTTT	87	33
30	88	0.115%	TGTGCTCTTGGGGAATGCCCCCCCTACTCTTGTCTCAAGCACAACTTGATCTGGGGGATACGCCCCGGGGAATTAGCTAAACTCATCTTT	90	45
31	193	0.076%	TGTGCTCTTGGGGAAAGAAGATTCCTAATGGTGGACTTCTTTGGGGGAAAACTGGGGGAACCGGACCGATACACCGATAAACTCATCTTT	90	40
38	185	0.109%	TGTGCTCTTGGGGAACCCCCCCCAGAGATTACGCCTGGGGGATACTCAAAGAAGTTCATCCAGAGGTCCAGGGGGCTGTACACCGATAAACTCATCTTT	99	52
32	60	0.050%	TGTGCTCTTGGGGAAGTCCCTGCCTCCTACTGGCCCGAGGATAAGAGGATGTTGGGGGAGACATACTCCCACTACACCGATAAACTCATCTTT	93	45
11	19	0.047%	TGTGCTCTTGGGGAACTAACTGGCAGATACACCTTCCTACGTGGCGGGGCCGGGGATACGGGCCTTTCGGGTACACCGATAAACTCATCTTT	93	37

Supplementary Figure 5: CDR3δ1 contain rare, unusually long functional sequences. A. Selected normalised spectratypes from two donors with notable extra-long CDR3 variants, shown with red circles, representative of 6 individuals. **B.** Selected well-represented extra-long TRDV1-TRDJ1 CDR3 sequences, showing actual read count in raw repertoire sequencing, % of total sequencing reads, overall length of CDR3 region (nt) and total N/P nucleotide addition in each sequence.

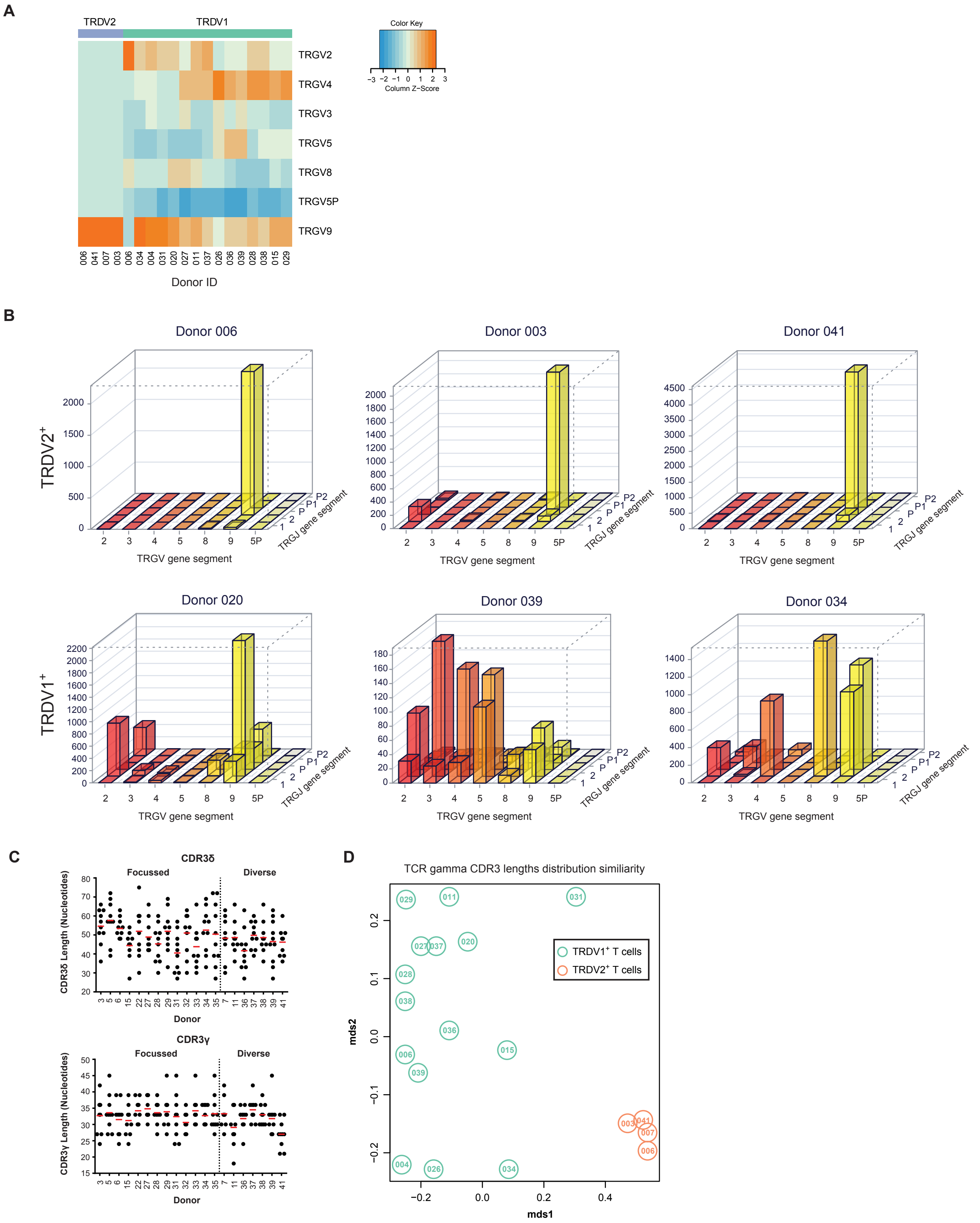

Supplementary Figure 6: TCRγ characteristics for Vδ1^{+} and Vδ2^{+} γδ T cells. A. Normalised Vγ gene segment usage in Vδ1^{+} and Vδ2^{+} γδ TCR repertoires. **B.** 3D representation of typical Vγ-Jγ gene segment usage in Vδ2^{+} (top row; showing 3 representative donors out of 4) and Vδ1^{+} (bottom row; showing 3 representative donors out of 20) γδ T cells, calculated per sequencing read (non-normalised). **C.** Length distribution and mean (red line) of the top 10 most prevalent clonotypes in CDR3δ (bottom left) and CDR3γ (bottom right) from indicated Vδ1^{+} T cell donors not shown in Fig 4D. **D.** Jensen-Shannon divergence between non-normalised TCRγ repertoires in Vδ1^{+} and Vδ2^{+} subsets based on the CDR3 amino acid sequence length, 2D representation of multidimensional clustering (VDJ Tools software).

A

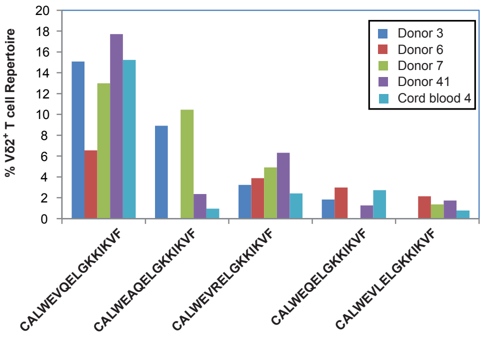

B

Donor 3	%	Donor 6	%	Donor 7	%	Donor 41	%	Cord blood 4	%
CALWEVQELGKKIKVF	15.1	CALWETQELGKKIKVF	22.9	CALWEVQELGKKIKVF	13.0	CALWEVQELGKKIKVF	17.7	CALWEVQELGKKIKVF	15.2
CALWEAQELGKKIKVF	8.9	CALWEVRKELGKKIKVF	18.5	CALWEAQELGKKIKVF	10.5	CALWEVRELGKKIKVF	6.3	CALWEQELGKKIKVF	2.7
CATWDGGYYKKLF	5.9	CALWEVQELGKKIKVF	6.6	CALWEVRELGKKIKVF	4.9	CALWEVFELGKKIKVF	5.5	CALWEVRELGKKIKVF	2.4
CALWEVRELGKKIKVF	3.2	CALWEVRELGKKIKVF	3.9	CASPELFYYKKLF	3.8	CALWEAGVLGKKIKVF	3.4	CATWDTTGWFKIF	1.5
CALWEGKQELGKKIKVF	2.1	CALWEVLSPQELGKKIKVF	3.4	CALWEVKELGKKIKVF	2.4	CALWEAQELGKKIKVF	2.4	CALWEAQELGKKIKVF	1.0
CALWEQELGKKIKVF	1.8	CALWEQELGKKIKVF	3.0	CALWQELGKKIKVF	1.8	CALWEVLELGKKIKVF	1.7	CALWEVELGKKIKVF	0.9
CALWTAQELGKKIKVF	1.7	CALWEVQGLGKKIKVF	2.3	CALWEVGELGKKIKVF	1.6	CALWSTELGKKIKVF	1.6	CALWEEELGKKIKVF	0.8
CALWEVHPGVFELGKKIKVF	1.4	CALWEVLELGKKIKVF	2.1	CALWEVLELGKKIKVF	1.4	CALWEVPELGKKIKVF	1.6	CALWEVLELGKKIKVF	0.8
CALWETSWELGKKIKVF	1.4	CALWENPKLGKKIKVF	2.0	CALWEVHELGKKIKVF	1.2	CALWEVHRELGKKIKVF	1.6	CATWDRNYYKKLF	0.8
CALWEEELGKKIKVF	1.4	CALWEVEELGKKIKVF	1.7	CALWEVEELGKKIKVF	1.2	CALWEQELGKKIKVF	1.3	CALWELGKKIKVF	0.8

C

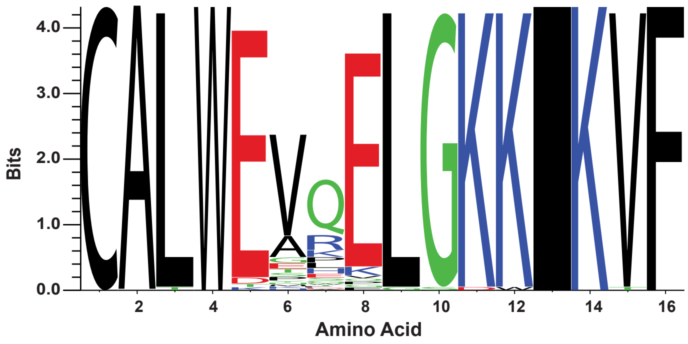

D

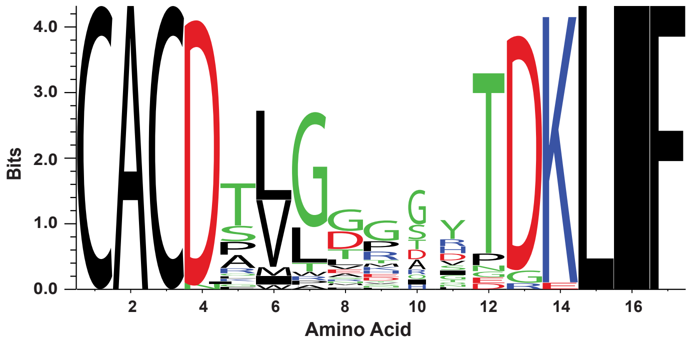

Supplementary Figure 7: Public Vγ9 sequences from Vδ2+ γδ T cells. A and B. Analysis of frequency **(A)** and sequence identity **(B)** of CDR3γ sequences from sorted Vδ2+ γδ T cells from four healthy adult donors and one cord blood. **C and D.** Visual representation of amino acid enrichment at each position of CDR3γ **(C)** and CDR3δ **(D)** sequences from Vδ2+ γδ T cells. Analysis was confined to the 20 most abundant CDR3γ of 14 amino acids from each of 4 adult donors, or ten most abundant CDR3δ2 sequences using Vδ2-Jδ1 of 13-15 amino acids length.

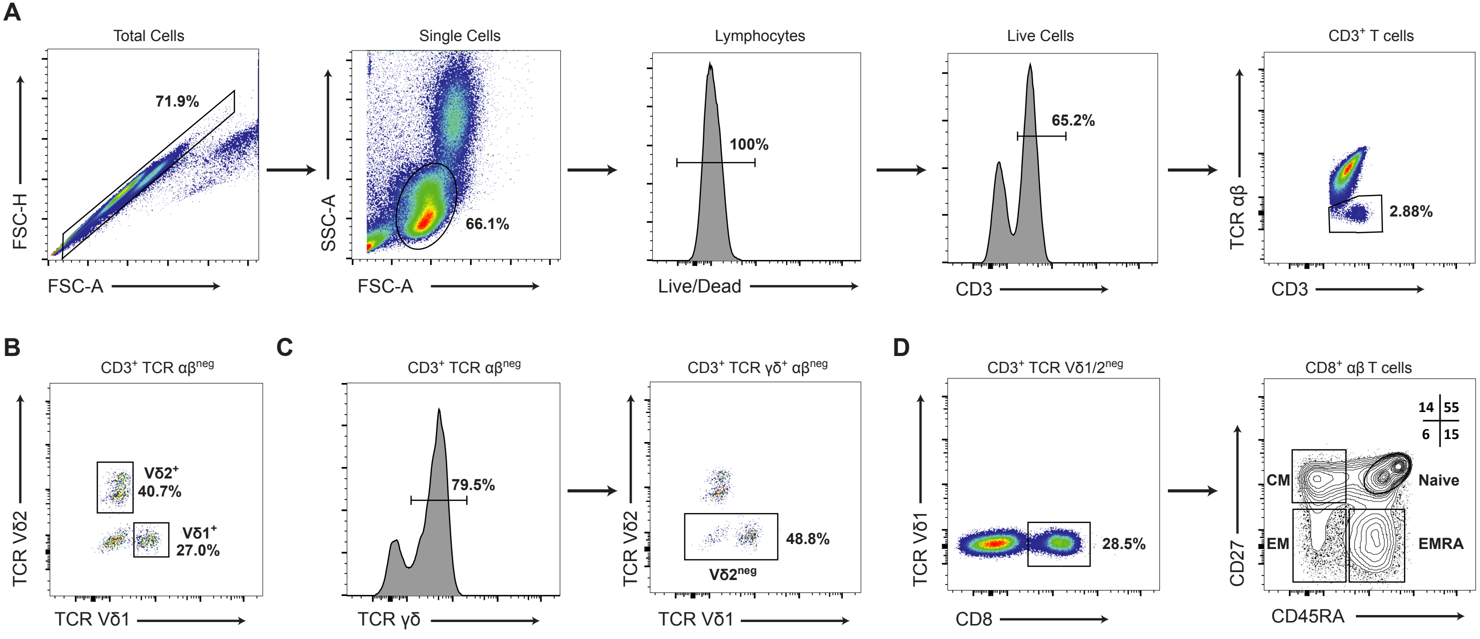

Supplementary Figure 8: Gating strategy for identifying and sorting T cells. A. Representative flow cytometry plots show the gating strategy used to identify single/live/lymphocytes and CD3^{+} TCR αβ^{neg} T cells. **B** and **C.** Using the gating strategy in **A.**, Vδ1^{+} and Vδ2^{+} (**B**) and Vδ2^{neg} γδ T cells (**C**) were sorted with TCR specific antibodies from CD3^{+} TCR αβ^{neg} T cells. **D.** CD8^{+} αβ T cells were identified by gating single/live/lymphocytes and CD3^{+} T cells (as shown in **A.**), selecting TCR Vδ1/Vδ2^{neg} cells and gating CD8^{+} T cells (left). CD27 and CD45RA were used to define memory T cell populations (Naïve, central memory; CM, effector memory; EM and effector memory CD45RA-revertants; EMRA) within CD8^{+} αβ T cells (right). Flow cytometry plots are representative of 20 donors.

Donor	TCR Chain	Sequencing Reads	Total CDR3	Unique CDR3	Cell Numbers (x 10^{3})
3	TRD	247354	215495	936	25
	TRG	489350	420583	657	25
5	TRD	301692	270866	1042	7.6
	TRG	249187	227705	643	7.6
6	TRD	188791	161794	359	7.2
	TRG	294125	284516	289	7.2
7	TRD	164806	140173	2650	25
	TRG	455352	396695	1789	25
11	TRD	193606	175261	936	25
	TRG	227924	208569	372	25
15	TRD	324650	308838	1676	25
	TRG	267362	260771	780	25
20	TRD	395517	371202	2051	25
	TRG	437885	415557	816	25
22	TRD	143487	142436	684	25
	TRG	389641	342630	390	25
26	TRD	359679	349164	7004	25
	TRG	562324	536932	3023	25
27	TRD	481992	457103	5443	25
	TRG	687234	635982	2826	25
28	TRD	110158	102974	2230	25
	TRG	125384	107150	563	25
29	TRD	288125	266648	1985	25
	TRG	611184	599058	1195	25
31	TRD	375423	350112	2253	25
	TRG	669944	645027	1356	25
32	TRD	149139	134080	1339	25
	TRG	463039	449407	928	25
33	TRD	366993	355469	2804	25
	TRG	341720	308335	1273	25
34	TRD	402725	354764	1156	25
	TRG	439176	403286	587	25
35	TRD	285454	284419	717	25
	TRG	599028	563321	497	25
36	TRD	379719	359488	10111	25
	TRG	346823	309217	3341	25
37	TRD	414550	385585	3763	25
	TRG	214117	185311	1522	25
38	TRG	147841	131069	1988	25
	TRD	429142	395512	5140	25
39	TRG	186273	179643	2758	25
	TRD	366753	345628	7117	25
41	TRG	538556	472281	804	25
	TRD	655818	636919	1665	25
CB01	TRD	133686	124265	2248	7.1
	TRG	252978	225747	1662	7.1
CB03	TRD	265021	254917	4696	11.4
	TRG	205191	188284	2085	11.4
CB04	TRD	298887	284049	6613	25
	TRG	181574	157254	3467	25
CB05	TRG	203485	189804	920	7
	TRD	336556	326726	1827	7
CB06	TRG	500765	430652	1066	5.2
	TRD	483213	471771	3433	5.2
31-CD27hi	TRG	457510	417978	1018	5
	TRD	579591	559376	2604	5
31-CD27lo	TRG	642840	628370	236	6.2
	TRD	578810	551791	384	6.2
29-CD27hi	TRG	436866	409659	1425	4
	TRD	501569	476057	3492	4
29-CD27lo	TRG	597053	534932	229	6
	TRD	516806	495834	118	6

Supplementary Table 1: Details of raw sequencing data. The table displays each sample's analysed total sequencing reads, total CDR3 assigned, unique CDR3 identified and the number of cells obtained per sample.

		TRGV	N/P	TRGJ		CDR3 length	N/P nts	CMV status	EBV status	D75
germline	TRGV2	GCC ACC TGG GAC GGG		G AAT TAT TAT AAG AAA CTC	TRGJ1/2					
	TRGV3/5	GCC ACC TGG GAC AGG		T GGG CAA GAG TTG GGC AAA AAA ATC AAG GTA	TRGJP					
	TRGV4	GCC ACC TGG GAT GGG		AT ACC ACT GGT TGG TTC AAG ATA	TRGJP1					
	TRGV8	GCC ACC TGG GAT AGG		AT AGT AGT GAT TGG ATC AAG ACG	TRGJP2					
	TRGV9	GCC TTG TGG GAG GTG								
clone										
POS4	TRGV8	GCC ACC TGG GAT A		CC ACT GGT TGG TTC AAG ATA	TRGJP1	33	0			
LES	TRGV4	GCC ACC TGG GAT GG	ATT	T TAT TAT AAG AAA CTC	TRGJ1/2	33	3			
donor										
29	TRGV2	GCC ACC TGG GAC GG	ATCC	TAT TAT AAG AAA CTC	TRGJ1/2	33	4	-	+	0.25
37	TRGV2	GCC ACC TGG GAC G	CCCTCGGG	TAT TAT AAG AAA CTC	TRGJ1/2	36	8	+	-	13.40
7	TRGV3	GCC ACC TGG GAC AGG	CGACCCAA	T AGT GAT TGG ATC AAG ACG	TRGJP2	42	8	-	+	16.00
28	TRGV3	GCC ACC TGG GAC AGG	ATTTT	T TAT AAG AAA CTC	TRGJ1/2	33	5	-	+	1.47
39	TRGV3	GCC ACC TGG GAC AGG	CCTG	AG AAA CTC	TRGJ1/2	27	4	+	+	28.80
27	TRGV4	GCC ACC TGG GAT GGG	CGTGAGGG	T TAT AAG AAA CTC	TRGJ1/2	36	8	+	+	11.73
6	TRGV5	GCC ACC TGG	TTCCTGGTAGG	G AAA CTC	TRGJ1/2	27	11	+	+	2.30
31	TRGV5	GCC ACC TGG G	TAC	AT AAG AAA CTC	TRGJ1/2	24	3	+	-	0.12
3	TRGV9	GCC TTG TGG G	GAAACCT	T TAT TAT AAG AAA CTC	TRGJ1/2	33	7	+	+	1.23
5	TRGV9	GCC TTG TGG GAG	CGGGG	G TTG GGC AAA AAA ATC AAG GTA	TRGJP	39	5	+	+	3.79
11	TRGV9	GCC TTG T	CTCAGCGGTCC	AAT TAT TAT AAG AAA CTC	TRGJ1/2	36	11	-	+	8.24
15	TRGV9	GCC TTG TGG	AGCCACCC	T TAT TAT AAG AAA CTC	TRGJ1/2	33	8	+	+	2.78
22	TRGV9	GCC TTG TGG GAG GTG	GCCGGGC	AT TAT AAG AAA CTC	TRGJ1/2	36	7	-	+	0.14
32	TRGV9	GCC TTG TGG GAG GTG		TAT TAT AAG AAA CTC	TRGJ1/2	30	0	-	+	0.07
33	TRGV9	GCC TTG TGG GAG GTG	CAA	ACT GGT TGG TTC AAG ATA	TRGJP1	36	3	-	+	1.87
34	TRGV9	GCC TTG TGG GAG G	ACTCG	TAT TAT AAG AAA CTC	TRGJ1/2	33	5	+	+	0.30
35	TRGV9	GCC TTG TGG GAG GTG	CATATTGGA TC	G AAT TAT TAT AAG AAA CTC	TRGJ1/2	45	11	+	+	0.11
36	TRGV9	GCC TTG T	CCCCTTC	T AAG AAA CTC	TRGJ1/2	24	7	-	-	27.21
38	TRGV9	GCC TTG TGG GAG G	G	G AAT TAT TAT AAG AAA CTC	TRGJ1/2	33	1	-	+	25.51
41	TRGV9	GCC TTG TGG GA		T TAT TAT AAG AAA CTC	TRGJ1/2	27	0	-	-	9.18
					average	33.15	5.8			
					range	24-45	0-11			

Supplementary Table 2: Prevalent CDR3γ sequences are private. The most prevalent TCRγ clonotype sequence from each adult donor is shown. Vγ and Jγ gene segments used are indicated, and N and P nucleotides are shown in red and blue, respectively. The CDR3γ length, number of N/P nucleotides, CMV/EBV status, and TCRγ diversity (D75) of each donor is shown.

		TRDV	N/P	TRDD1	TRDD2	N/P	TRDD3	N/P	TRDJ		CDR3 length	N/P nts	CMV status	EBV status	D75
germline	TRDV1	GCT CTT GGG GAA CT		GAAATAGT	CCTTCCTAC		ACTGGGGGATACG		AC ACC GAT AAA CTC ATC	TRJD1					
	TRAV29/DV5	GCA GCA AGC G							CT TTG ACA GCA CAA CTC TTC	TRJD2					
clone															
POS4	TRDV1	GCT CTT GGG GAA CT					GGGGGAT		GAT AAA CTC ATC	TRJD1	33	0			
LES	TRDV5	GCA GCA AGC	TCC		CCTA	TTA	GGGGATA	TACAGGGT	CC GAT AAA CTC ATC	TRJD1	48	14			
donor															
3	TRDV1	GCT CTT GGG GAA C			CTAC	CTCATC GT	ACTGGGGGATAC	TAGGGGGA	ACC GAT AAA CTC ATC	TRJD1	60	16	+	+	1.32
5	TRDV1	GCT CTT GGG GAA	GTGCGAT				ACTGGGGGATAC	AGCC	A CTC ATC	TRJD1	42	11	+	+	3.88
6	TRDV1	GCT CTT GGG GA	CCTC				CTGGGGGATACG	CCAGTACTTCATA	AC ACC GAT AAA CTC ATC	TRJD1	57	17	+	+	3.14
7	TRDV1	GCT CTT GGG GA	CGG		CCTTCC	CT	ATACG	GAAGGGGTGTTGT	AC ACC GAT AAA CTC ATC	TRJD1	57	18	-	+	12.4
11	TRDV1	GCT CTT GGG G	TAAAACGGGGT				CTGGGGGATAC	TCAAG	AA CTC ATC	TRJD1	45	16	-	+	9.86
15	TRDV1	GCT CT	CCAACCCC		CCTAC	G		CGCTGCCTGT	C ACC GAT AAA CTC ATC	TRJD1	45	19	+	+	5.12
22	TRDV1	GCT CTT GGG GA	CGCAGATT		TTCCTA	GATGGAG	ACTGGG	CCACC	AC ACC GAT AAA CTC ATC	TRJD1	60	20	-	+	0.12
27	TRDV1	GCT CTT	ATCTCACT				TGGGGGATACG	CT	ACC GAT AAA CTC ATC	TRJD1	42	10	+	+	4.02
28	TRDV1	GCT CTT GGG GA	CACCCGGCCTCCGAG		CCTAC	GTT	ACTGGGGG	CTCAG	C GAT AAA CTC ATC	TRJD1	60	23	-	+	5.12
29	TRDV1	GCT CTT GGG GAA C	AT		TTCC	ATACCGTT GT	ACTGGGGGATACG		GAT AAA CTC ATC	TRJD1	54	12	-	+	1.33
31	TRDV1	GCT CTT GGG GAA C	A		TCCT	CCGT	ACTGGGG	GATCTCC	CT TTG ACA GCA CAA CTC TTC	TRJD2	57	13	+	-	0.38
32	TRDV1	GCT CTT GGG	TTGCC GG		CCTTCCTA	TAC	ACTGGG	TTCG	CC GAT AAA CTC ATC	TRJD1	51	14	-	+	0.82
33	TRDV1	GCT CTT GGG GAA C					CTGGGGGAT	TCGAG	CTC ATC	TRJD1	33	5	-	+	3.25
34	TRDV1	GCT CTT GGG GAA	AGGCGGATCGCA		TTCCTAC	GA	GGGATACG		C ACC GAT AAA CTC ATC	TRJD1	57	14	+	+	0.41
35	TRDV1	GCT CTT GGG GAA C	CCGGAGGCCT				TGGGG	CCCA	C GAT AAA CTC ATC	TRJD1	45	14	+	+	0.11
36	TRDV1	GCT CTT GGG GAA CT	CGGAG						AA CTC ATC	TRJD1	27	5	-	-	24.6
37	TRDV1	GCT CTT GGG	ACTCCC		CCTTC	AAGGGTC	GGGGGATA	TTCAGG	T AAA CTC ATC	TRJD1	51	19	+	-	11.6
38	TRDV1	GCT CTT GGG GA	TCTA		TTCC		ACTGGGGGATAC	TGGCGCATCGTCCCAACTC GT	AC ACC GAT AAA CTC ATC	TRJD1	69	25	-	+	17
39	TRDV1	GCT CTT GGG GAA CT	GGTCATCGGACC				CTGGGGGATAC	ACCCCCG	C ACC GAT AAA CTC ATC	TRJD1	60	19	+	+	24.2
41	TRDV1	GCT CTT GGG	AACCCGGGAGA		CCTTCCTA	ACT	ACTGGG	AAACGT	AC ACC GAT AAA CTC ATC	TRJD1	60	20	-	-	14.5
										average	51.6	15.5			
										range	27-69nt	5-25nt			

Supplementary Table 3: Prevalent CDR3δ1 sequences are complex and private. The most prevalent TCRδ1 clonotype sequence from each adult donor. Sequences were analysed using IMGT Junction Analysis, which identified V, D, and J gene segments used, and highlighted N (red) and P (blue) nucleotides. The CDR3δ1 length, number of N/P nucleotides, CMV/EBV status of each donor, and TCRδ diversity (D75) is shown.

germline	TRGV		CDR3γ	TRGJ				
				NYYKKLF	TRGJ1/2			
	TRGV2-8	CATWDG		GQELGKKIKVF	TRGJP*01			
	TRGV9	CALWEV		TTGWFKIF	TRGJP1*01			
				SSDWIKTF	TRGJP2*01			
clone						CDR3 length(aa)	CMV status	EBV status
POS4	TRGV8	CATWD		TTGWFKIF	TRGJP1*01	11	+	?
LES	TRGV4	CATWDG	F	YYKKLF	TRGJ1/2	11	+	?
donor								
29	TRGV2	CATWDG	S	YYKKLF	TRGJ1/2	11	-	+
37	TRGV2	CATWD	ALG	YYKKLF	TRGJ1/2	12	+	-
7	TRGV3	CATWD	RRPN	SDWIKTF	TRGJP2*01	14	-	+
28	TRGV3	CATWD	RIF	YKKLF	TRGJ1/2	11	-	+
39	TRGV3	CATWD	RPE	KLF	TRGJ1/2	9	+	+
27	TRGV4	CATWDG	REG	YKKLF	TRGJ1/2	12	+	+
6	TRGV5	CATW	FLVG	KLF	TRGJ1/2	9	+	+
31	TRGV5	CATW	VH	KKLF	TRGJ1/2	8	+	-
3	TRGV9	CALW	GNL	YYKKLF	TRGJ1/2	11	+	+
5	TRGV9	CALWE	RG	LGKKIKVF	TRGJP*01	13	+	+
11	TRGV9	CAL	SQRS	NYYKKLF	TRGJ1/2	12	-	+
15	TRGV9	CALW	SHP	YYKKLF	TRGJ1/2	11	+	+
22	TRGV9	CALWEV	AGH	YKKLF	TRGJ1/2	12	-	+
32	TRGV9	CALWEV		YYKKLF	TRGJ1/2	10	-	+
33	TRGV9	CALWEV	Q	TGWFKIF	TRGJP1*01	12	-	+
34	TRGV9	CALWE	DS	YYKKLF	TRGJ1/2	11	+	+
35	TRGV9	CALWEV	HIGS	NYYKKLF	TRGJ1/2	15	+	+
36	TRGV9	CAL	SPS	KKLF	TRGJ1/2	8	-	-
38	TRGV9	CALWE	GNL	NYYKKLF	TRGJ1/2	11	-	+
41	TRGV9	CALW	D	YYKKLF	TRGJ1/2	9	-	-
					average (aa)	11.05		
					range	8-15aa		

Supplementary Table 4: Prevalent CDR3γ sequences are private. The most prevalent TCRγ clonotype sequence from each adult donor. Sequences were analysed using IMGT Junction Analysis, which identified V, D, and J gene segments used. The CDR3γ length, and CMV/EBV status of each donor is shown.

germline		TRDV	CDR3δ	TRDJ				
				TDKLIF	TRDJ1			
	TRDV1	CALGE		LTAQLFF	TRDJ2			
	TRDV5	CAAS		SWDTRQMFF	TRDJ3			
				RPLIF	TRDJ4			
clone						CDR3 length(aa)	CMV status	EBV status
POS4	TRDV1	CALGE	LGD	DKLIF	TRJD1	11	+	?
LES	TRDV5	CAAS	SPIRGYTGS	DKLIF	TRJD1	16	+	?
donor								
3	TRDV1	CALGE	PTSSYWGILGG	TDKLIF	TRJD1	20	+	+
5	TRDV1	CALGE	VRYWGIQP	LIF	TRJD1	14	+	+
6	TRDV1	CALG	DLLGDTPVLHN	TDKLIF	TRJD1	19	+	+
7	TRDV1	CALG	DGLPYTEGVLY	TDKLIF	TRJD1	19	-	+
11	TRDV1	CALG	VKRGLGDTQE	LIF	TRJD1	15	-	+
15	TRDV1	CAL	QPPYALPV	TDKLIF	TRJD1	15	+	+
22	TRDV1	CALG	DADFPRWRLGHH	TDKLIF	TRJD1	20	-	+
27	TRDV1	CAL	ISLGGYA	TDKLIF	TRJD1	14	+	+
28	TRDV1	CALG	DTRPPSLRYWGLS	DKLIF	TRJD1	20	-	+
29	TRDV1	CALGE	HFHTVVLGD	TDKLIF	TRJD1	18	-	+
31	TRDV1	CALGE	HPPYWGDLP	LTAQLFF	TRJD2	19	+	-
32	TRDV1	CALG	LPAFLYTGFA	DKLIF	TRJD1	17	-	+
33	TRDV1	CALGE	PGGFE	LIF	TRJD1	11	-	+
34	TRDV1	CALGE	RRIAFLRGIR	TDKLIF	TRJD1	19	+	+
35	TRDV1	CALGE	PGGLGAH	DKLIF	TRJD1	15	+	+
36	TRDV1	CALGE	LGE	LIF	TRJD1	9	-	-
37	TRDV1	CALG	TPPSRVGGYSG	KLIF	TRJD1	17	+	-
38	TRDV1	CALG	DLFHWGILAHRPNSY	TDKLIF	TRJD1	23	-	+
39	TRDV1	CALGE	LVIGPWGIHPR	TDKLIF	TRJD1	20	+	+
41	TRDV1	CALG	NPGDLPNYWETY	TDKLIF	TRJD1	20	-	-
					average	17.2		
					range	9-23aa		

Supplementary Table 5: Prevalent CDR3δ1 sequences are complex and private. The most prevalent TCRδ1 clonotype sequence from each adult donor. Sequences were analysed using IMGT Junction Analysis, which identified V, D, and J gene segments used. The CDR3δ1 length (amino acids), and CMV/EBV status of each donor is shown
